# A new genus and nine new species of Eugnomini (Coleoptera, Curculionidae) from New Caledonia

**DOI:** 10.3897/zookeys.554.6120

**Published:** 2016-01-18

**Authors:** Miłosz A. Mazur

**Affiliations:** 1 Center for Biodiversity Studies, Department of Biosystematics, Opole University, Oleska 22; 45–052 Opole, Poland

**Keywords:** Coleoptera, Curculionidae, Curculioninae, Eugnomini, Rasilinus, new genus, new species, New Caledonia, distribution, key to species

## Abstract

The genus *Rasilinus* **gen. n**. is described (type species *Rasilinus tchambicus* sp. n.). Nine new species: *Rasilinus bicolor* **sp. n**., *Rasilinus bifurcatus* **sp. n.**, *Rasilinus bimaculatus* **sp. n.**, *Rasilinus grandidens* **sp. n**., *Rasilinus longulus* **sp. n.**, *Rasilinus subgemellus* **sp. n.**, *Rasilinus subnodulus* **sp. n.**, *Rasilinus tchambicus* **sp. n.**, *Rasilinus virgatus* **sp. n.** are described from New Caledonia. Illustrations of the external morphology, male and female terminalia, dorsal habitus colour photographs of the adults, key to species and distribution map of the new genus *Rasilinus* are provided.

## Introduction

The Eugnomini is a small, poorly studied tribe of the speciose family Curculionidae. The classification, biogeography, and biology of most species are poorly known. Currently, Eugnomini comprises about 200 known species included in 28 genera and is primarily an Old World group occurring in New Zealand (19 genera), Australia (five), New Caledonia (three) and New Guinea (one) (Zimmerman 1994; Alonso-Zarazaga and Lyal 1999; Macfarlane et al. 2011; Kuschel 2014; Mazur 2014a, b). In the New World the tribe is represented by four genera known from South America; only one genus, *Rhopalomerus* Blanchard, occurs both in the New and Old Worlds (Alonso-Zarazaga and Lyal 1999). More detailed study about the tribe with general diagnosis and systematic background were presented last time by Cawthra (1966). The newest checklist of all known genera, data on their distribution and comments on the current classification of the Eugnomini were presented by Mazur (2014b). Detailed summary history of New Caledonian fauna with zoogeographic and evolutionary implications, based on apionid weevils (Coleoptera: Curculionoidea: Apionidae), were presented by Wanat (2001).

This paper presents descriptions of a new genus and nine species of Eugnomini from New Caledonia along with a key to all new species.

## Materials and methods

This study is based on 37 specimens. Holotypes are deposited in the Muséum National d’Histoire Naturelle, Paris (MNHN). Paratypes are deposited in institutions abbreviated below. Abbreviations are used throughout the paper:

DBUO
 Department of Biosystematics, Opole University, Poland 


MNHW
Museum of Natural History, University of Wrocław, Poland 


NZAC
New Zealand Arthropod Collection, Lincoln, New Zealand 


QM
Queensland Museum, Australia 


USMB
 Natural History Department of Upper Silesian Museum, Bytom, Poland 


Measurements were taken using a calibrated stereomicroscopic grid eyepiece (C–W10xB/22) and stereomicroscope NIKON SMZ–800. Genitalia preparations were made according to the standard method of macerating the separated abdomen for 5–10 minutes in a hot KOH solution. Photographs were taken using the camera a CANON Power Shot A640 camera connected with the stereomicroscope and processed using the Helicon Focus v. 4.50 and PhotoFiltre v. 6.1 software programmes.

The nomenclature of the male terminalia and abbreviations of particular measurements (partly modified) follows Wanat (2001) (alphabetical order):

apw pronotum width at anterior margin;

arw width of rostrum apex;

bew width of elytral base (measured through the middle of humeral calli);

bpw pronotum width at the base;

el elytra length, measured in top view in a position when the base and apex of elytra are at the same level;

eyl eye length, measured in top view, when the head is positioned horizontally;

frw minimum frons width;

hl head length;

hw head width, measured across the middle of the eyes;

lb length of body exclusive of rostrum;

mew maximum elytra width;

mith minimum height of tooth on hind femur, measured from basal part of femur (shorter edge);

mpw maximum pronotal width;

pl pronotum length;

ptbl protibia length;

ptbmw maximum width of fore tibia;

mth maximum height of tooth on hind femur; measurement from apex of femur (longer edge);

rl rostrum length, measured in top of view, when base and apex of rostrum are in the same level;

scl antennal scape length.

The nomenclature of antennal parts follows Lyal (http://weevil.info/glossary-weevil-characters).

All dimensions are given in millimetres.

## Taxonomic treatment

### Rasilinus gen. n.

Taxon classificationAnimaliaColeopteraCurculionidae

http://zoobank.org/E5E03CF2-1CB8-4854-9455-72E4E80B3C62

#### Type species.

*Rasilinus tchambicus* sp. n. Gender masculine.

#### Diagnosis.

Body massive; elytra narrowed, smooth, without any tubercles or spines, not covered with adjacent scales, sculpture of elytra clearly visible; hind femora stout, strongly clavate with large tooth, their basal part stout.

#### Description.

Size 2.5–3.6 mm. Body stout, distinctly convex transversally. Rostrum dorsoventrally flattened, as long as head or slightly longer; dorsal surface covered with white, more or less protruding, elongate scales, sometimes mixed with additional white scales which are suboval and adjoining. Antennae inserted on apical part of rostrum; funicle with seven antennomeres; scape reaching posterior margin of eye. Scrobe deep in lateral view with sharp edges; partly visible in dorsal view of rostrum; not passing along ventral part and not reaching front margin of eyes. Eyes weakly to strongly convex. Frons narrower than base of rostrum. Pronotum subquadrate. Elytra widest at basal half, distinctly and regularly narrowed to apex; apically truncate or rounded; humeral calli weakly or strongly prominent, posterior calli absent. Surface of elytra glabrous, more or less shining; intervals wider than striae with single line of short, protruding, inclined backward setae; entire surface in some species covered with extremely small, hardly visible, piliform setae. Second pair of wings well developed. Front coxae contiguous. Trochanters small, fusiform. Posterior margin of metaventrite with distinct line of dense, closely adjoining, white scales. All femora covered with sparse, elongate scales. Outer margin of tibiae covered with white, elongate scales. Hind femora broadened, clavate with enlarged tooth; dorsal surface with contrasting, transverse stripe of white scales (except *Rasilinus subnodulus* sp. n.); posterior margin of tooth on hind femora with two types of piliform setae: one short and straight and other elongate and hooked apically. Hind tibiae strongly curved. Segments of front and middle tarsi similar in length; first tarsal segment of hind tarsi elongate, as long as 2+3 or longer. Male pygidium (tergite VIII) with two narrow processes on ventral side. Tegmen with elongate or very short parameres. Penis well sclerotised, most species with fully sclerotised base of pedon (here, basal part of pedon is the anterior ventral margin of the penis body between the apodemes) and variable, well visible structures in internal sac.

#### Etymology.

The generic name is derived from the Latin adjective *rasilis* (smooth) and refers to the elytral sculpture. Gender masculine.

#### Distribution.

**Map 1.**
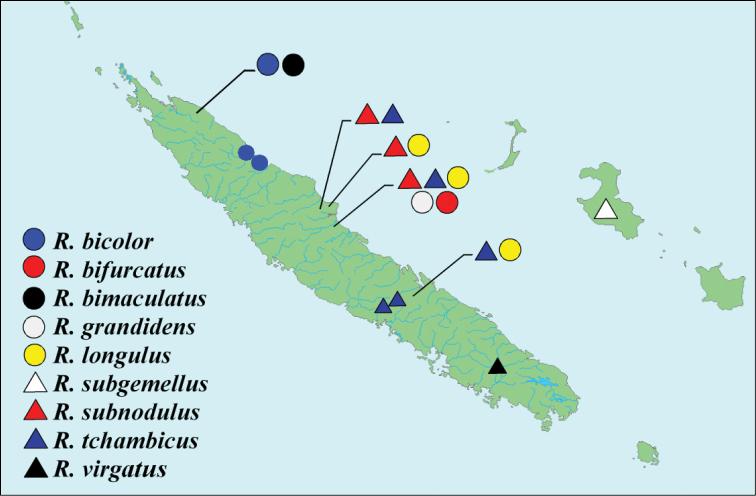
Distribution of species from genus *Rasilinus* gen. n. in New Caledonia.

New Caledonia (main island), only *Rasilinus subgemellus* sp. n. is known from Lifou Island (Map 1).

#### Biology.

Detailed biology unknown. Other members of Eugnomini have been reared from dead wood, subcortical tissues, live stems, galls, leaves or fruits of many species of plants in different families (e.g. May 1987).

### Rasilinus tchambicus sp. n.

Taxon classificationAnimaliaColeopteraCurculionidae

http://zoobank.org/27621B98-059F-4457-B97D-A2221039B182

Figs 8 , 17 , 26 , 35 , 44 , 70‒73 , 84 , 91 , 98 , 105 , 112 , 119 , 125 , 130 , 135

#### Diagnosis.

One of the smallest members of new genus. The following combination of characters allows this species to be distinguished from its congeners: rostrum short and relatively thin with compare to its congeners, almost as long as head; pronotum wider than long; elytra weakly narrowed; minute tooth on fore and middle femora; apodemes much longer than aedegal pedon; tegmen with completely separated parameroid lobes.

#### Description.


 Body length (lb) *ca.* 2.50 mm.

*Body colour and vestiture* (Fig. 8). Colour of body variable, from uniformly light brown to almost black. Elytra, pronotum and head usually dark brown, except for lighter rostrum and legs. Legs with three possible types of coloration: i) dark femora with lighter tibiae, ii) light femora (sometimes with darkening base) with darker tibiae (sometimes only apical part darkening), iii) femora and tibiae with the same colour. Rostrum and frons covered with two kinds of white scales: one suboval and recumbent and other elongate, protruding. Outer margin of tibiae with white, elongate, protruding scales. Elytral intervals with single line of protruding, elongate scales with variable colour, from dark on basal to bright on apical part of elytra. Entire surface of elytra covered with additional minute, hardly visible, short, piliform, recumbent scales.

**Figures 1–9.**
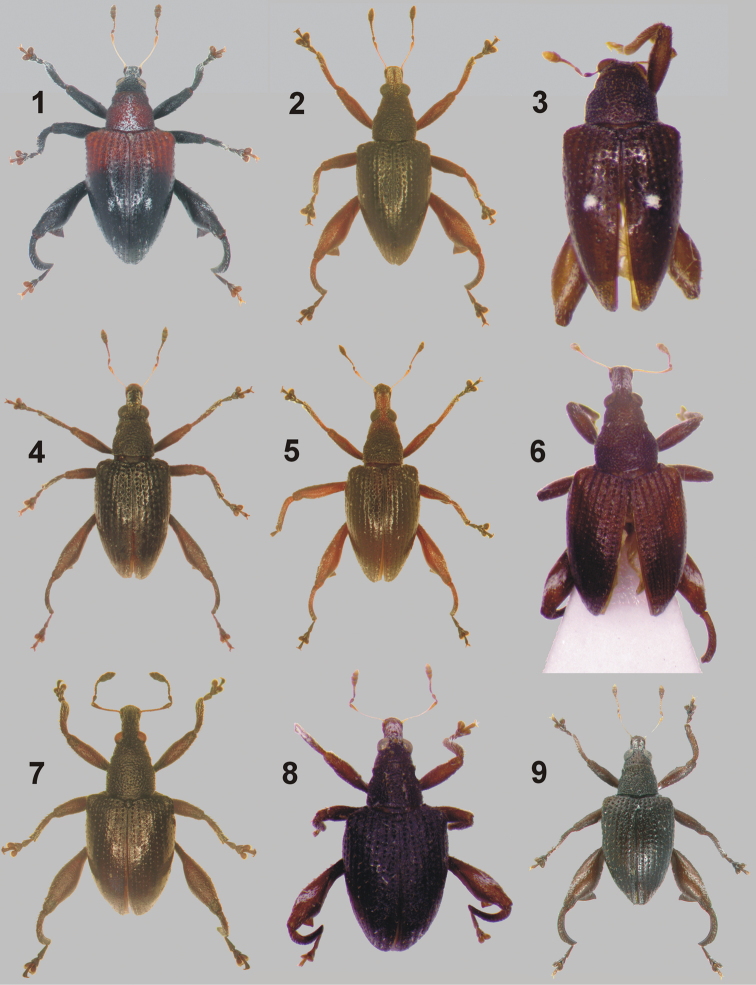
Dorsal habitus colour photographs of New Caledonian species from the genus *Rasilinus* gen. n. **1** *Rasilinus bicolor* sp. n., paratype, ♀, Mandjélia (MNHW) **2** *Rasilinus bifurcatus* sp. n., holotype, ♂, Auopinié, (MNHN) **3** *Rasilinus bimaculatus* sp. n., holotype, ♂, Mandjélia (MNHN) **4** *Rasilinus grandidens* sp. n., holotype, ♂, Auopinié (MNHN) **5** *Rasilinus longulus* sp. n., paratype, ♀, Pic d’Amoa (QM) **6** *Rasilinus subgemellus* sp. n. holotype, ♂, Lifou Is. (MNHN) **7** *Rasilinus subnodulus* sp. n., holotype, ♂, Auopinié (MNHN) **8** *Rasilinus tchambicus* sp. n., paratype, ♀, Tchamba (DBUO) **9** *Rasilinus virgatus* sp. n., holotype, ♂, Dzumac Mts. (MNHN).

**Figures 10–18.**
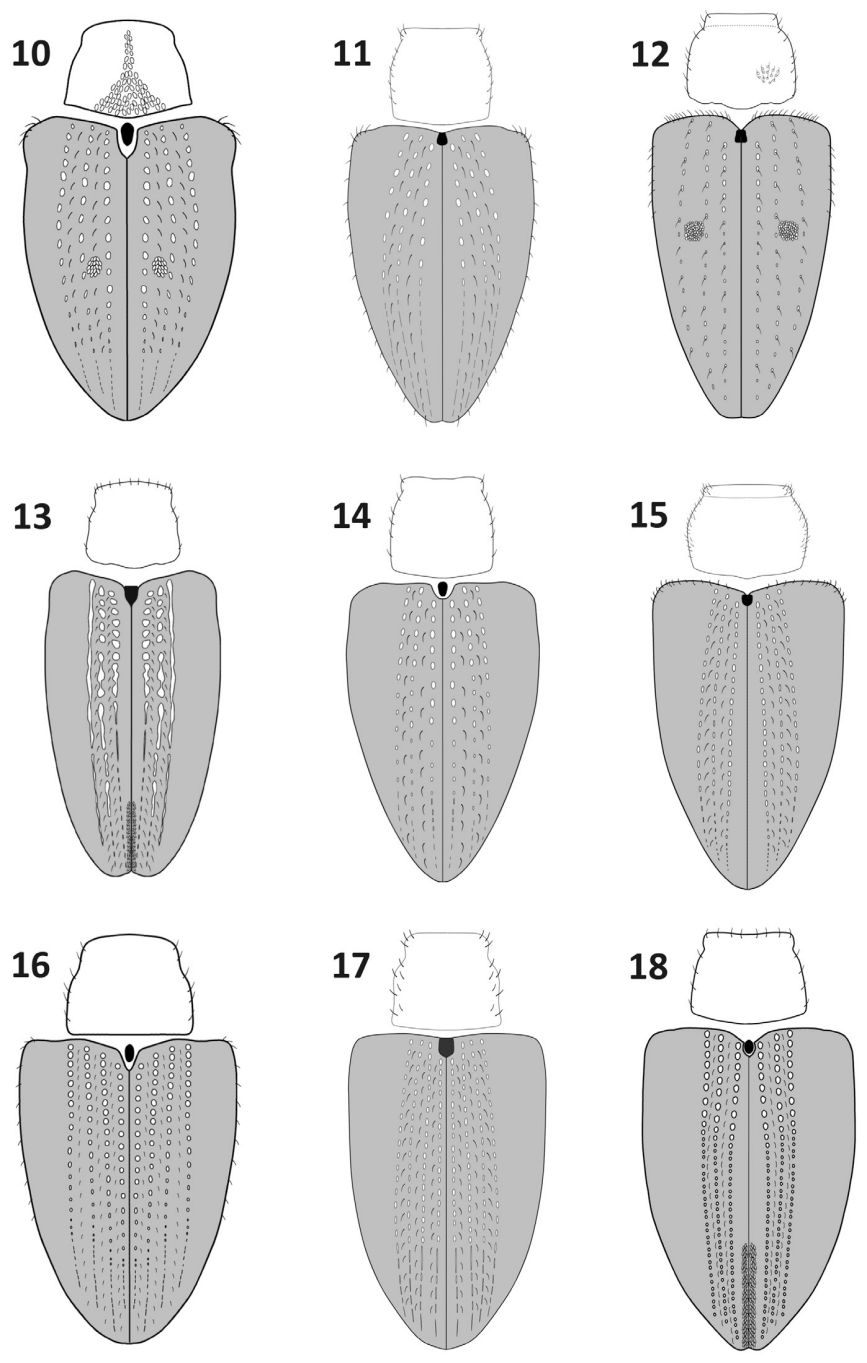
Elytra and pronotum, dorsal view: **10** *Rasilinus bicolor* sp. n. **11** *Rasilinus bifurcatus* sp. n. **12** *Rasilinus bimaculatus* sp. n. **13** *Rasilinus grandidens* sp. n. **14** *Rasilinus longulus* sp. n. **15** *Rasilinus subgemellus* sp. n. **16** *Rasilinus subnodulus* sp. n. **17** *Rasilinus tchambicus* sp. n. **18** *Rasilinus virgatus* sp. n.

*Head, rostrum and antennae* (Figs 26, 35, 44). Head elongate (hw/hl 1.30–1.45). Forehead flat. Eyes distinctly convex laterally; protruding above margin of head in lateral view; longer than half length of head (eyl/hl 0.63–0.70). Lateral margin of head narrowed from its base to hind margin of eyes. Vertex strongly scabrous, matt. Rostrum longer than width at apex (rl/arw 1.20–1.40), almost straight. Funicle much shorter than scape; first desmomere as long as 2 and 3 combined; desmomeres 4–7 subcircular, wider than long. Club *ca.* 2 × as long as its wide, longer than desmomeres 2–7.

**Figures 19–27.**
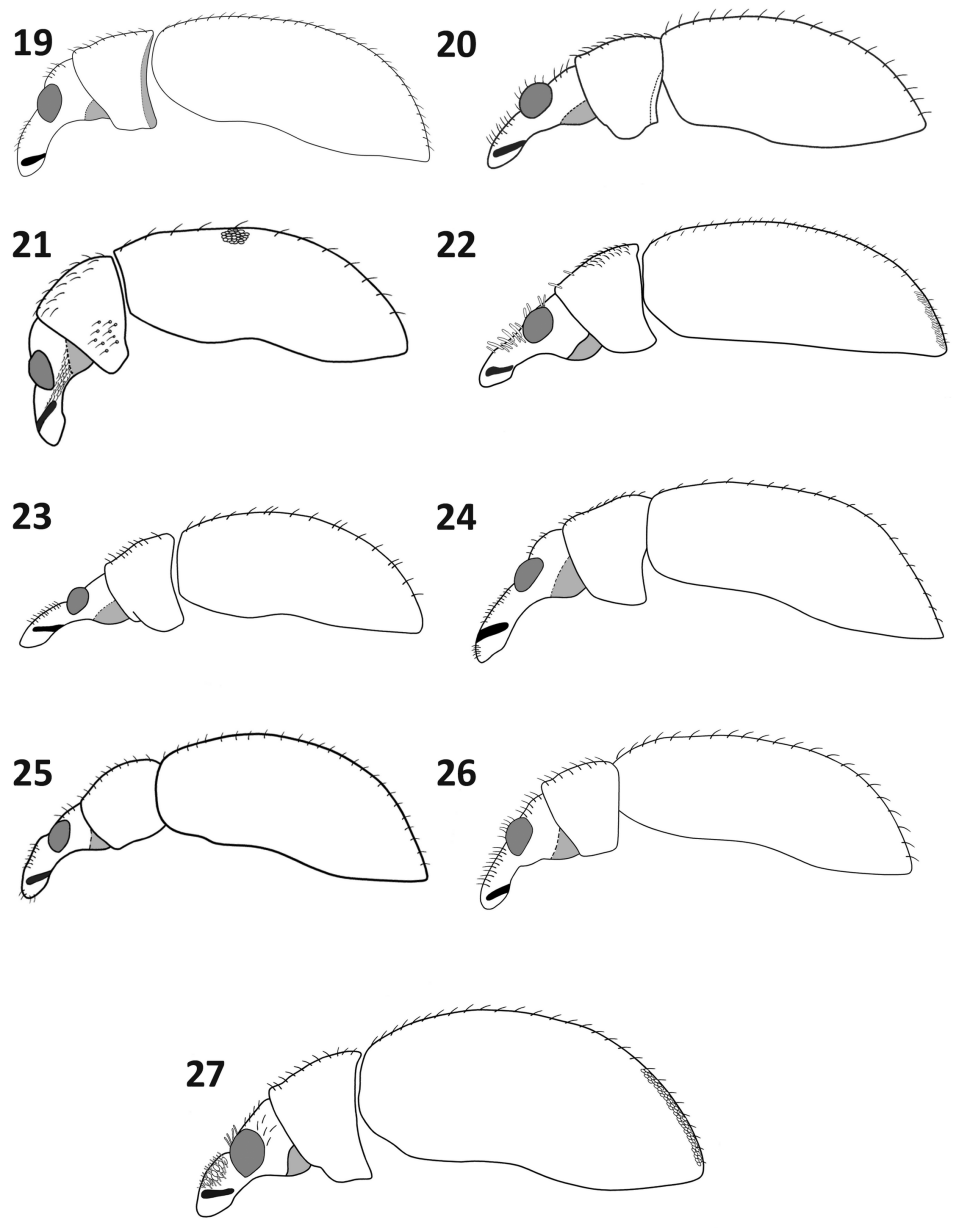
General habitus, lateral view: **19** *Rasilinus bicolor* sp. n. **20** *Rasilinus bifurcatus* sp. n. **21** *Rasilinus bimaculatus* sp. n. **22** *Rasilinus grandidens* sp. n. **23** *Rasilinus longulus* sp. n. **24** *Rasilinus subgemellus* sp. n. **25** *Rasilinus subnodulus* sp. n. **26** *Rasilinus tchambicus* sp. n. **27** *Rasilinus virgatus* sp. n.

**Figures 28–36.**
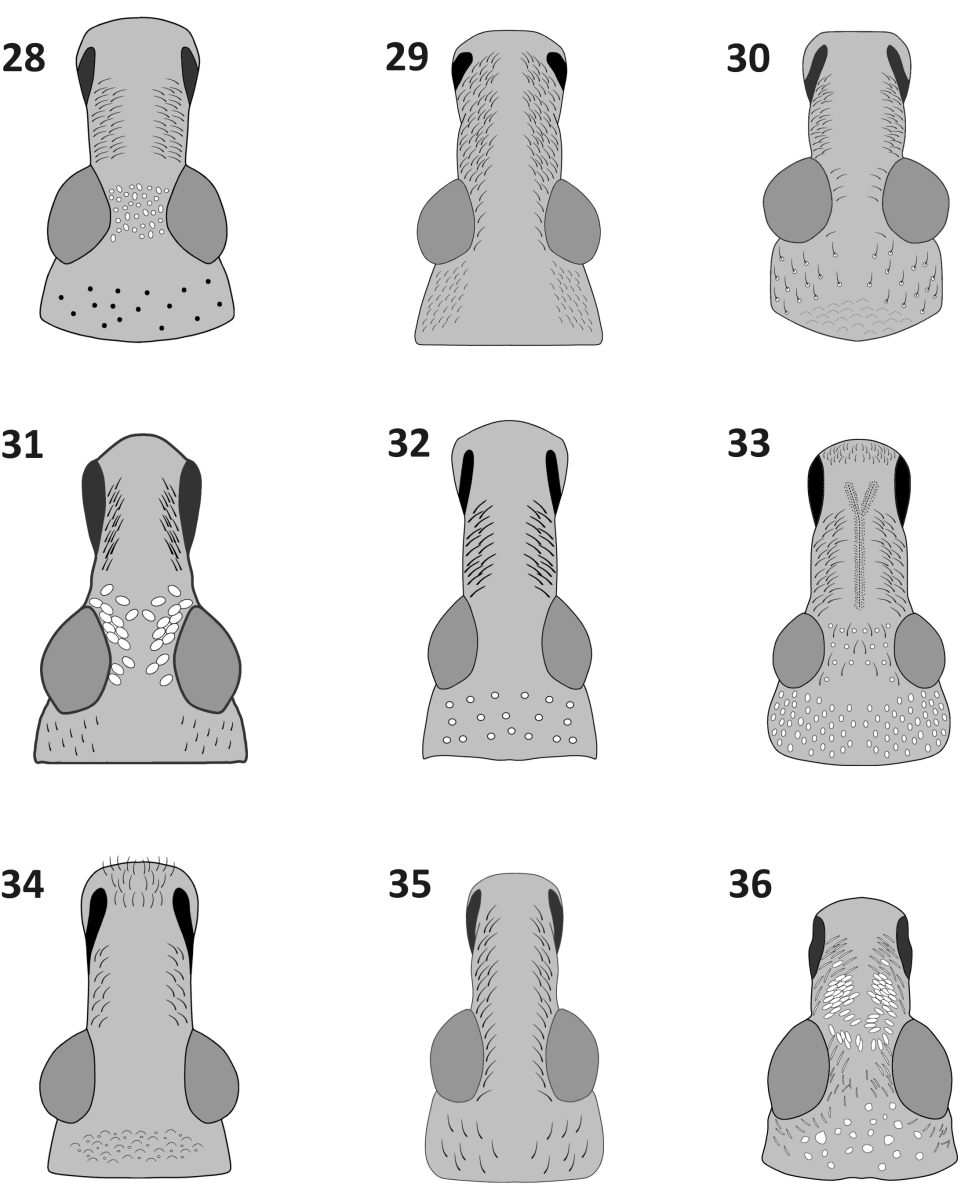
Head and rostrum, dorsal view: **28** *Rasilinus bicolor* sp. n. **29** *Rasilinus bifurcatus* sp. n. **30** *Rasilinus bimaculatus* sp. n. 31 *Rasilinus grandidens* sp. n. **32** *Rasilinus longulus* sp. n. **33** *Rasilinus subgemellus* sp. n. **34** *Rasilinus subnodulus* sp. n. **35** *Rasilinus tchambicus* sp. n. **36** *Rasilinus virgatus* sp. n.

**Figures 37–48.**
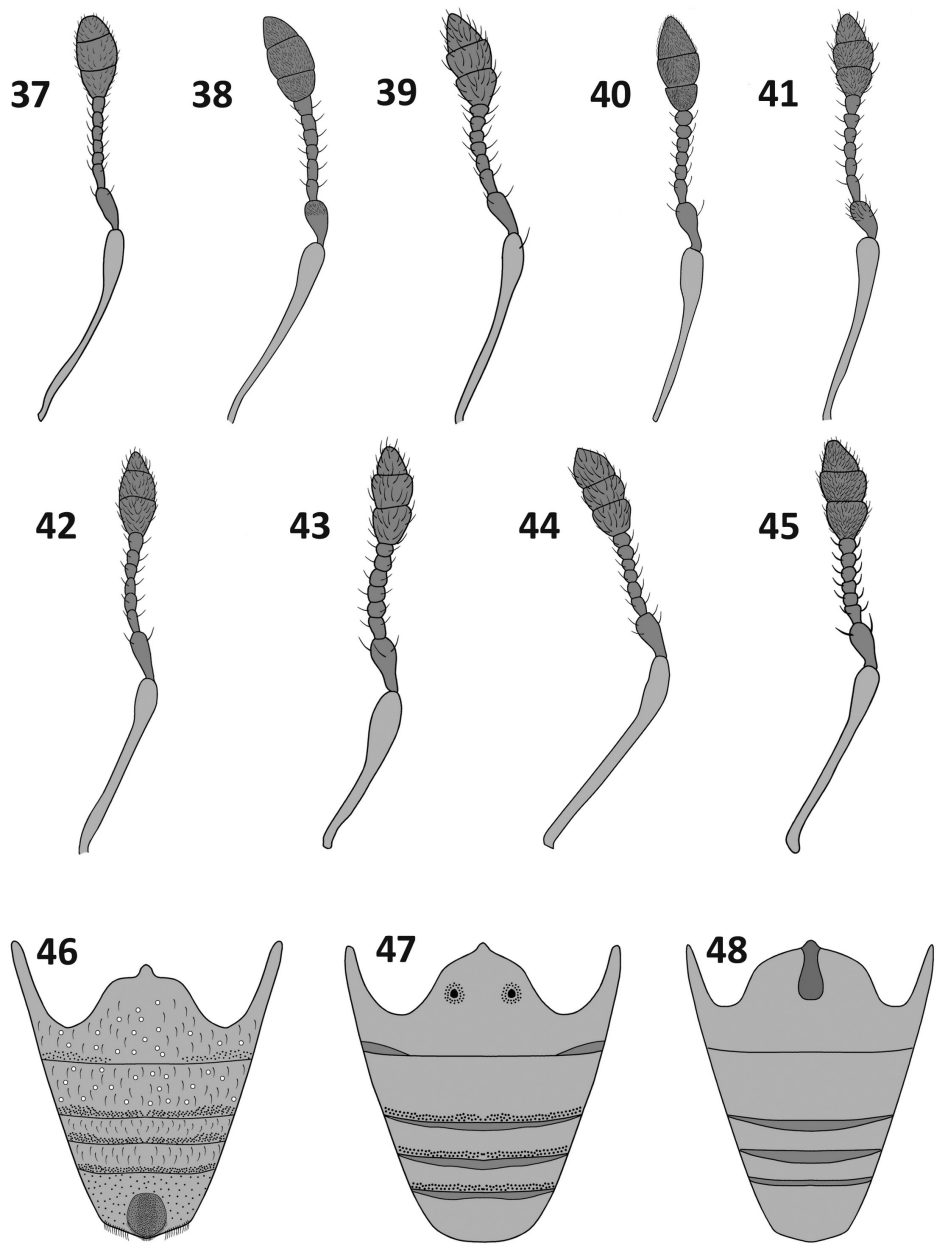
Antennae (**37–45**) and abdominal sternites (**46–48**): **37** *Rasilinus bicolor* sp. n. **38** *Rasilinus bifurcatus* sp. n. **39** *Rasilinus bimaculatus* sp. n. **40** *Rasilinus grandidens* sp. n. **41** *Rasilinus longulus* sp. n. **42** *Rasilinus subgemellus* sp. n. **43** *Rasilinus subnodulus* sp. n. **44** *Rasilinus tchambicus* sp. n. **45** *Rasilinus virgatus* sp. n. **46** *Rasilinus bimaculatus* sp. n. **47** *Rasilinus subgemellus* sp. n. **48** *Rasilinus subnodulus* sp. n.

**Figures 49–66.**
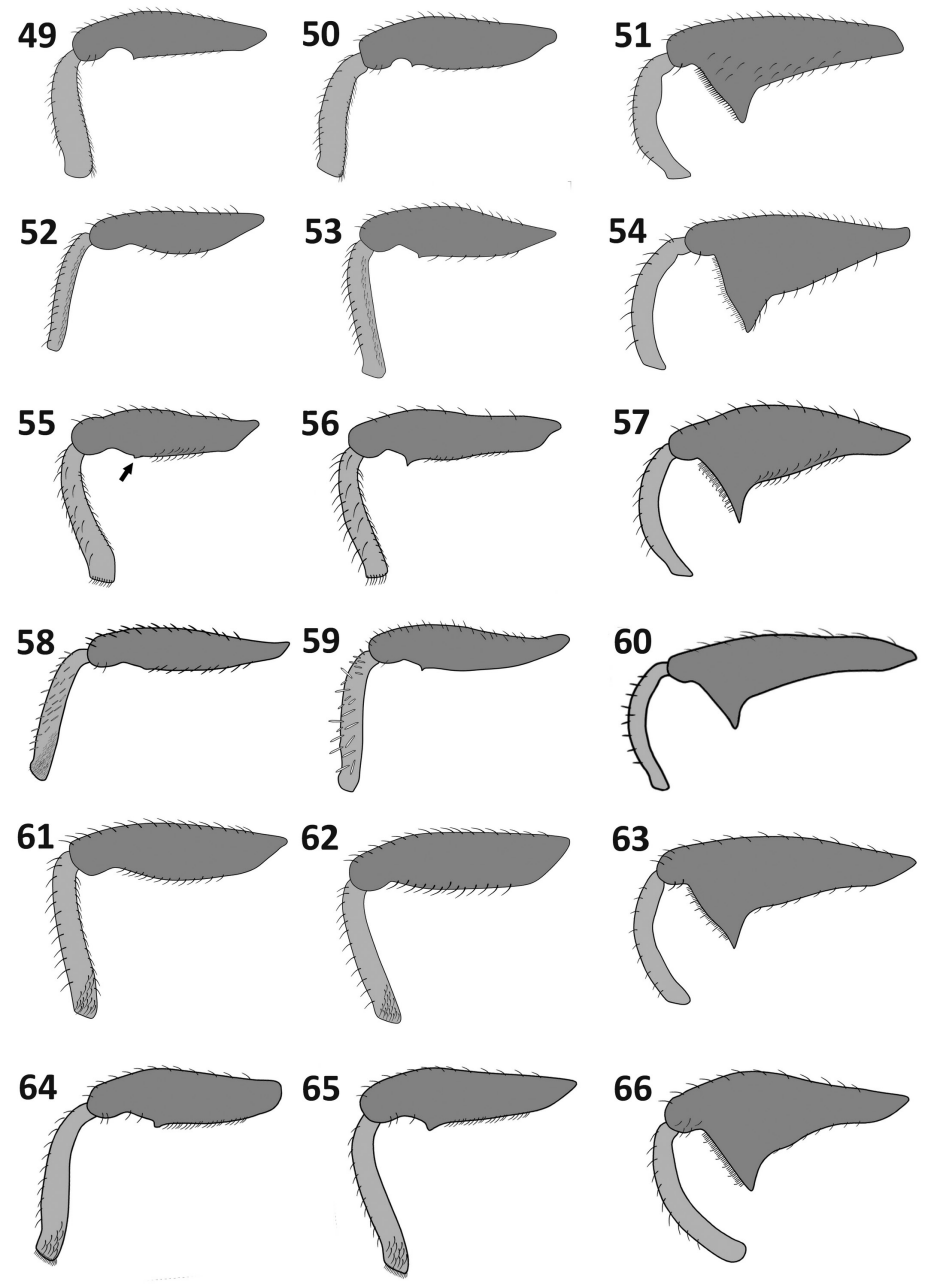
Legs: *Rasilinus bicolor* sp. n.: **49** front leg **50** middle leg **51** hind leg *Rasilinus bifurcatus* sp. n.: **52** front leg **53**–middle leg **54** hind leg *Rasilinus bimaculatus* sp. n.: **55** front leg (minute tooth marked by arrow) **56** middle leg **57** hind leg *Rasilinus grandidens* sp. n.: **58** front leg **59** middle leg **60** hind leg *Rasilinus longulus* sp. n.: **61** front leg **62** middle leg **63** hind leg *Rasilinus subgemellus* sp. n,: **64** front leg **65**–middle leg **66** hind leg.

*Pronotum* (Figs 17, 26). Wider than long (mpw/pl 1.15–1.27). Laterally slightly narrowed near base; strongly narrowed subapically. Apical margin straight; base slightly bisinuate. Surface distinctly rough, matt.

*Elytra* (Figs 17, 26). Elongate (el/mew 1.35–1.45), widest across humeral calli, weakly narrowed to apex. Striae with suboval, distinct punctures, at apical part of elytra punctures evanescent, forming indistinct line. Intervals glabrous, shining, weakly convex.

*Legs* (Figs 70–73, 84). Front and middle femora robust, with small teeth beyond middle; tibiae robust, front slightly sinuate, middle straight. Hind femora slightly narrowed beyond base; tibiae regularly curved, narrowed subapically. Claws regularly curved, distinctly broadened basally.

**Figures 67–85.**
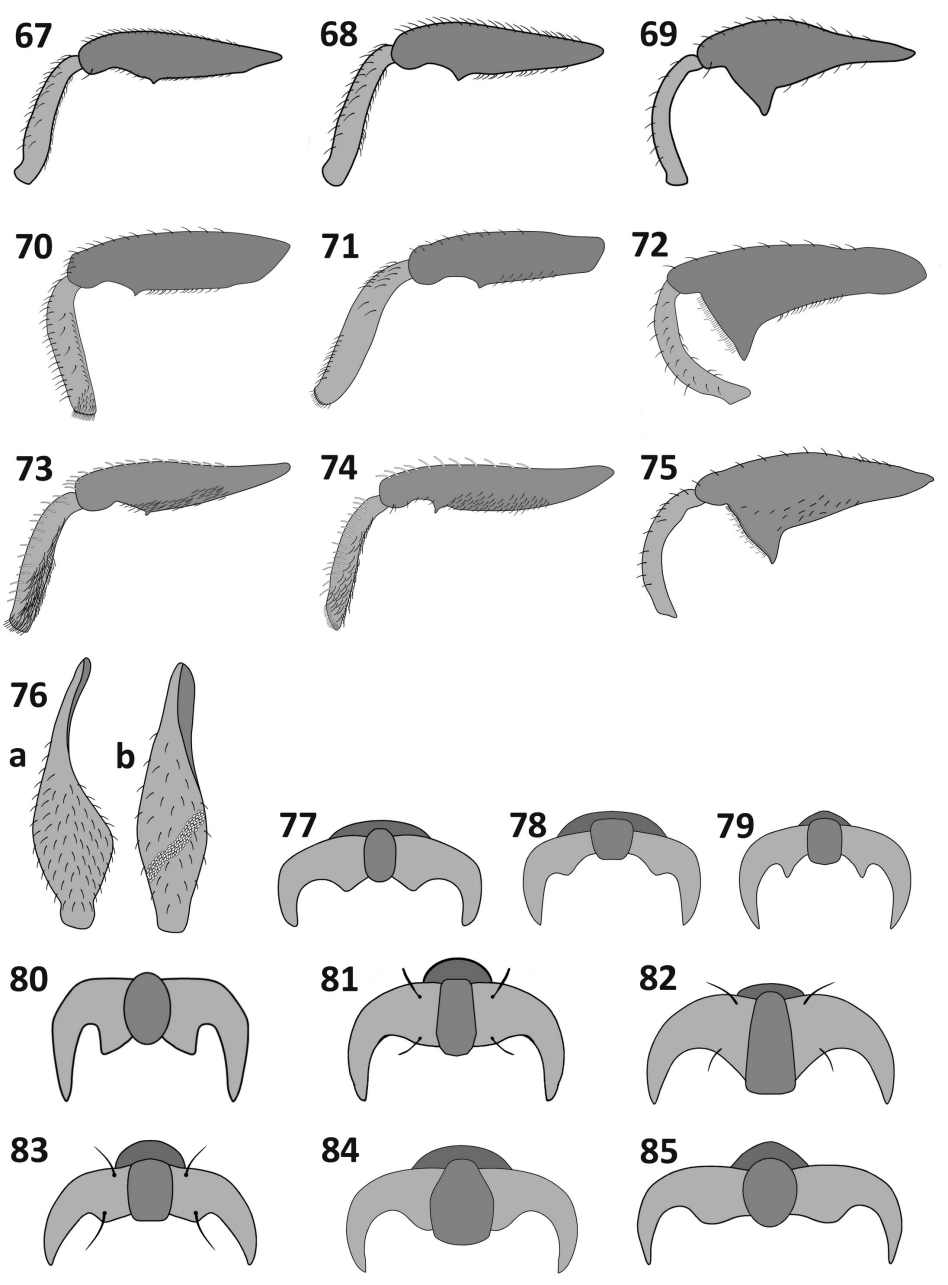
Legs (**67–75**) and tarsal claws (**77–85**): *Rasilinus subnodulus* sp. n.: **67** front leg **68** middle leg **69** hind leg *Rasilinus tchambicus* sp. n.: **70** front leg **71** middle leg **72** hind leg *Rasilinus virgatus* sp. n.: **73** front leg **74** middle leg **75** hind leg **76** hind femora, dorsal view of: a –*Pactola* (Pasc.) and b–*Rasilinus* gen. n. **77** *Rasilinus bicolor* sp. n. **78** *Rasilinus bifurcatus* sp. n. **79** *Rasilinus bimaculatus* sp. n. **80** *Rasilinus grandidens* sp. n. **81** *Rasilinus longulus* sp. n. **82** *Rasilinus subgemellus* sp. n. **83** *Rasilinus subnodulus* sp. n. **84** *Rasilinus tchambicus* sp. n. **85** *Rasilinus virgatus* sp. n.

*Abdomen* (Fig. 91). Short, 1.08 × longer than wide. First suture obsolete at middle of length. Sutures between ventrites 2–5 distinct, depressed. Last ventrite trapezoidal, almost 2 × as wide as long, apical margin almost straight. Pygidium of male subquadrate; apical margin almost straight with numerous, elongate setae; lobed middle part small with single setae.

**Figures 86–99.**
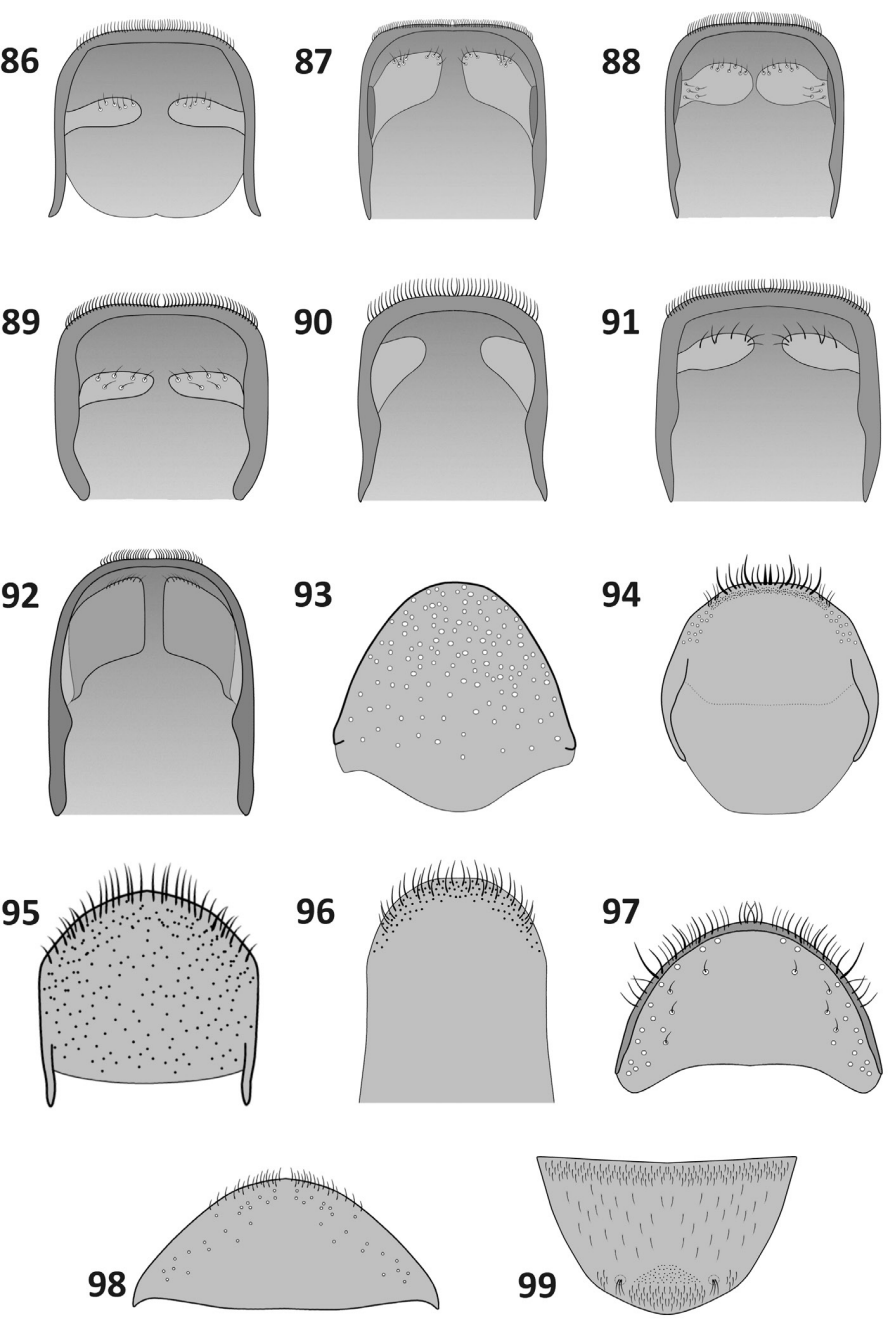
Terminalia. Male pygidium, ventral view (**86–92**), female pygidium, dorsal view (**93**), female abdominal tergite VIII, dorsal view (**94–98**) and last ventrite of male (**99**): **86** *Rasilinus bicolor* sp. n. **87** *Rasilinus bifurcatus* sp. n. **88** *Rasilinus bimaculatus* sp. n. **89** *Rasilinus subgemellus* sp. n. **90** *Rasilinus subnodulus* sp. n. **91** *Rasilinus tchambicus* sp. n. **92** *Rasilinus virgatus* sp. n. **93** *Rasilinus grandidens* sp. n. **94** *Rasilinus bicolor* sp. n. **95** *Rasilinus grandidens* sp. n. **96** *Rasilinus longulus* sp. n. **97** *Rasilinus subnodulus* sp. n. **98** *Rasilinus tchambicus* sp. n. **99** *Rasilinus bifurcatus* sp. n.

*Male terminalia* (Figs 105, 112, 119). Aedegal pedon shorter than apodemes; basal part sclerotised, slightly extended; lateral margins equilateral at basal half, from middle to apex distinctly narrowed; apex widely rounded. From lateral view distinctly, regularly curved with upturned apex. Endophallus not projecting outside pedon, internal structure not visible. Parameroid lobes of tegmen strongly elongate, thin, completely separated, of similar length to tegminal apodeme. Sternite VIII with bifurcate basal part; laterally flattened, apodeme slightly curved apically with thin apical keel; hemisternites on sternite IX subtriangular, clearly visible.

**Figures 100–113.**
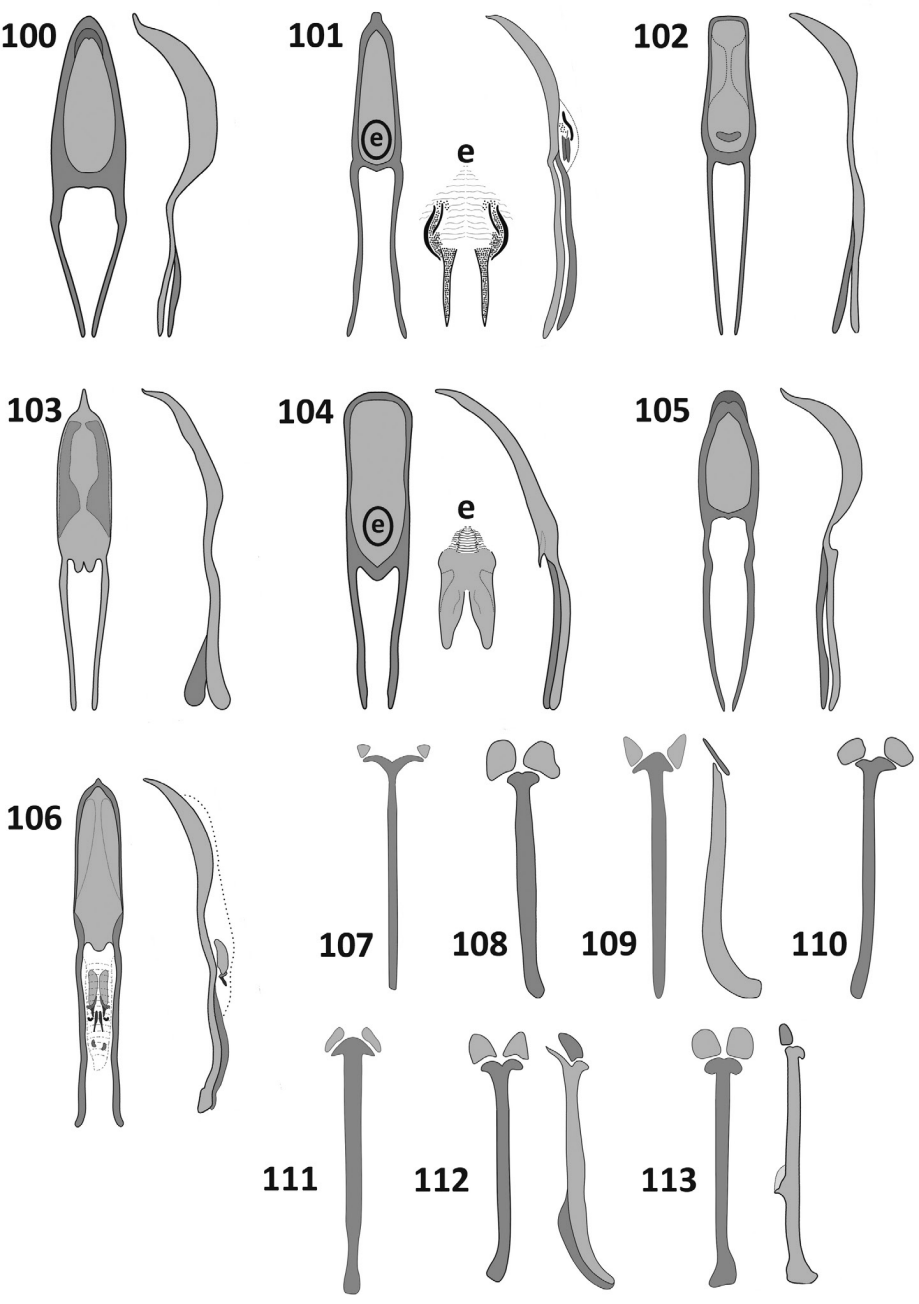
Male terminalia. Aedeagus, dorsal and lateral view (**100–106**) spiculum gastrale, dorsal view (**107, 108, 110, 111**) spiculum gastrale, dorsal and lateral view (**109, 112, 113**): **100** *Rasilinus bicolor* sp. n. **101** *Rasilinus bifurcatus* sp. n. (e–endophallus with characteristic sclerites) **102** *Rasilinus bimaculatus* sp. n. **103** *Rasilinus subgemellus* sp. n. **104** *Rasilinus subnodulus* sp. n. **105** *Rasilinus tchambicus* sp. n. **106** *Rasilinus virgatus* sp. n. **107** *Rasilinus bicolor* sp. n. **108** *Rasilinus bifurcatus* sp. n. **109** *Rasilinus bimaculatus* sp. n. **110** *Rasilinus subgemellus* sp. n. **111** *Rasilinus subnodulus* sp. n. **112** *Rasilinus tchambicus* sp. n. **113** *Rasilinus virgatus* sp. n.

**Figures 114–130.**
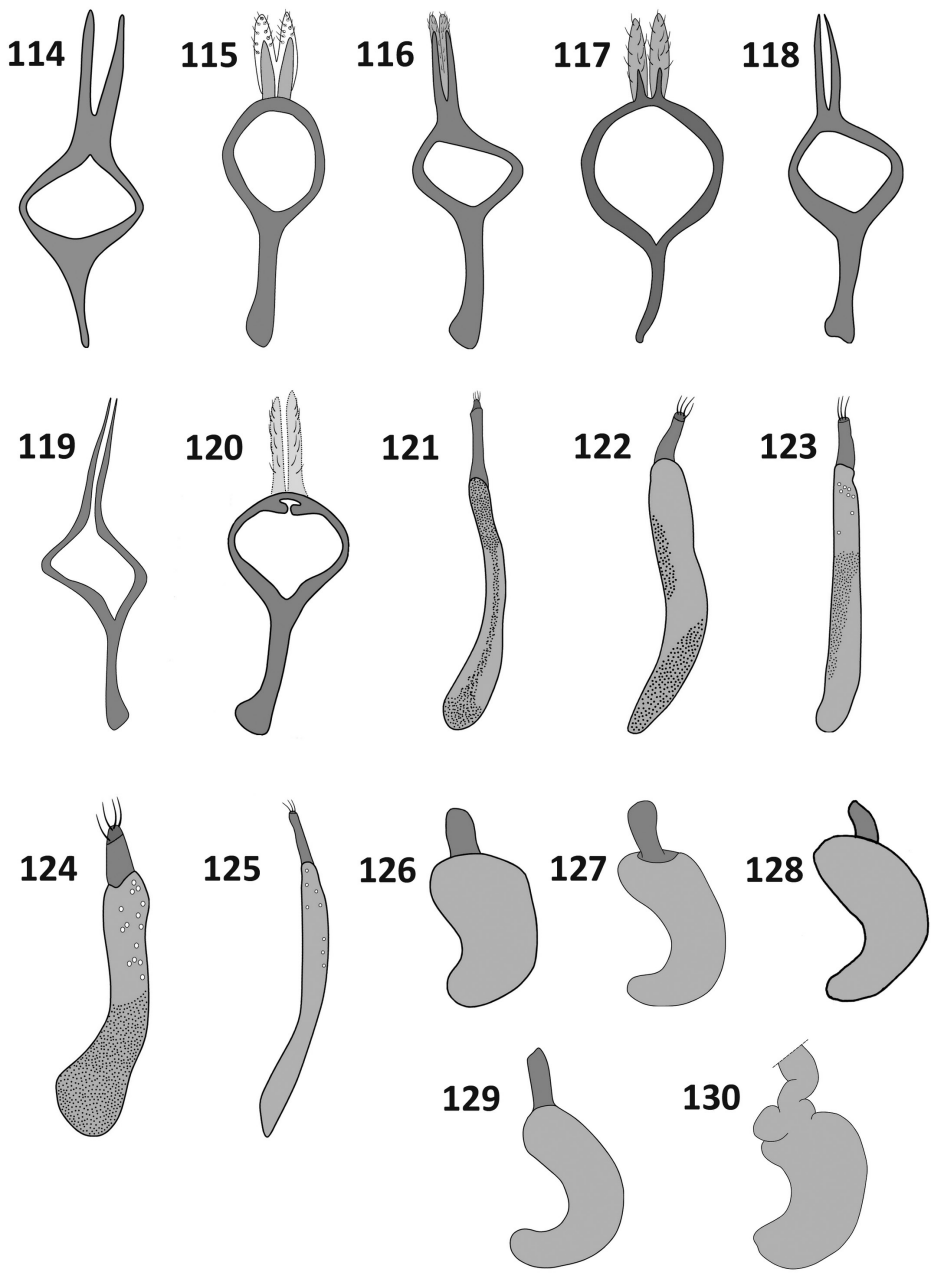
Terminalia. Male tegmen (**114–120**), female ovipositor (**121–125**) and spermatheca (**126–130**) : **114** *Rasilinus bicolor* sp. n. **115** *Rasilinus bifurcatus* sp. n. **116** *Rasilinus bimaculatus* sp. n. **117** *Rasilinus subgemellus* sp. n. **118** *Rasilinus subnodulus* sp. n. **119** *Rasilinus tchambicus* sp. n. **120** *Rasilinus virgatus* sp. n. **121** *Rasilinus bicolor* sp. n. **122** *Rasilinus grandidens* sp. n. **123** *Rasilinus longulus* sp. n. **124** *Rasilinus subnodulus* sp. n. **125** *Rasilinus tchambicus* sp. n. **126** *Rasilinus bicolor* sp. n. **127** *Rasilinus grandidens* sp. n. **128** *Rasilinus longulus* sp. n. **129** *Rasilinus subnodulus* sp. n. **130** *Rasilinus tchambicus* sp. n.

*Female* (Figs 98, 125, 130, 135). Similar to male in body shape but slightly larger. Elytra stout, widest beyond humeral calli, near middle. Apodeme of sternite VIII elongate, base bifurcate, irregular. Gonocoxite thin, elongate, more than 4 × as long as its wide; stylus minute with few setae. Spermatheca stout, distinctive curved. Abdominal tergite VIII more than 2 × wider than long; widely rounded apically; basally with slightly narrowed angles; surface with sparse punctation and few setae on apical margin.

**Figures 131–135.**
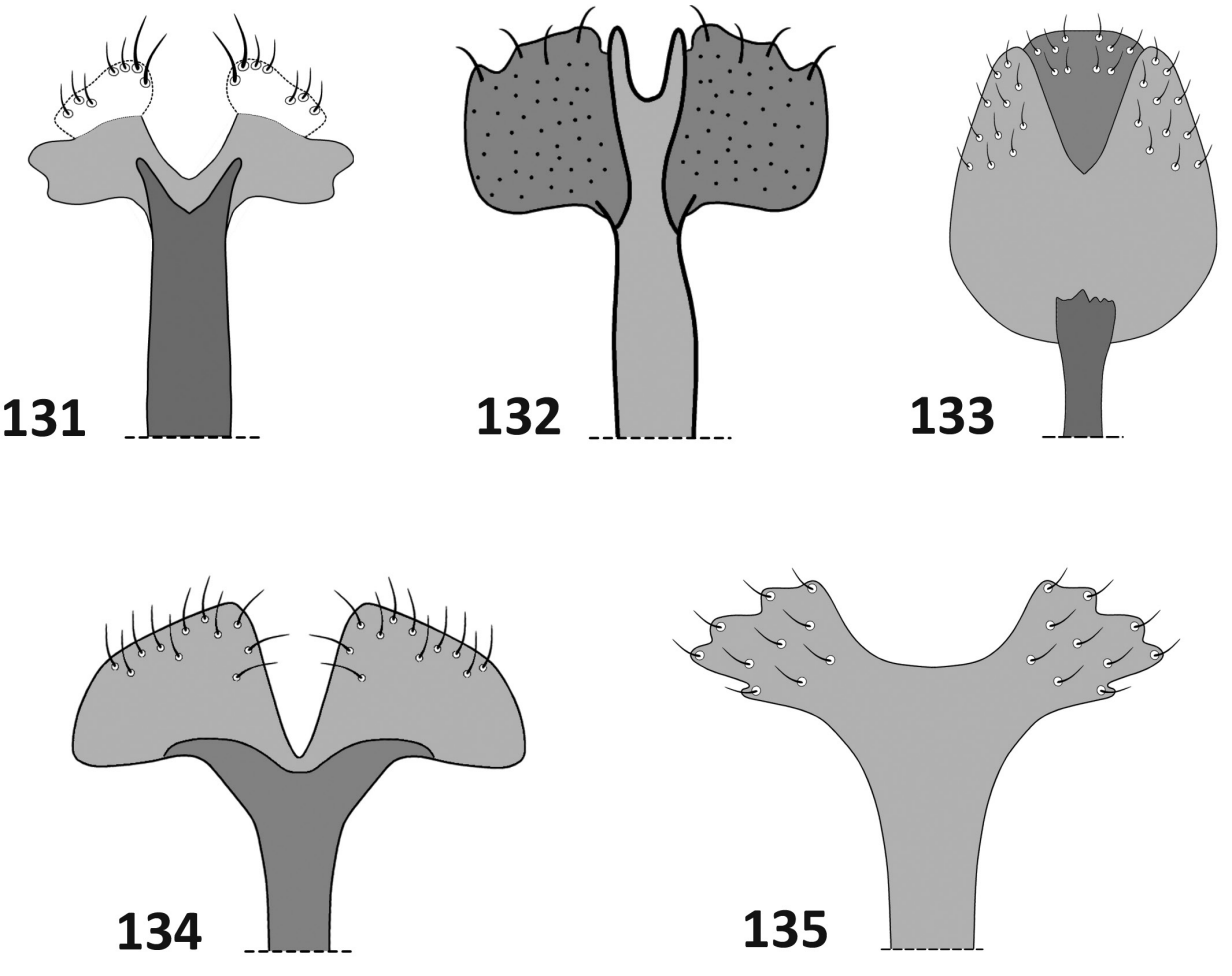
Terminalia. Female, apex of sternite VIII: **131** *Rasilinus bicolor* sp. n. **132** *Rasilinus grandidens* sp. n. **133** *Rasilinus longulus* sp. n. **134** *Rasilinus subnodulus* sp. n. **135** *Rasilinus tchambicus* sp. n.

#### Measurements.

Holotype, ♂: apw 0.45; arw 0.25; bew 1.10; bpw 0.60; el 1.50; eyl 0.25; frw 0.15; hl 0.35; hw 0.45; lb 2.45; mew 1.10; mith 0.15; mpw 0.60; mth 0.30; pl 0.50; ptbl 0.55; ptbmw 0.10; rl 0.35; scl 0.45.

Paratypes, ♂/♀: apw 0.45–0.50/0.55; arw 0.25–0.30/0.30; bew 1.10–1.25/1.30–1.35; bpw 0.60–0.70/0.70–0.75; el 1.50–1.80/1.90–2.00; eyl 0.25/0.25; frw 0.15/0.15; hl 0.35–0.40/0.35; hw 0.35–0.45/0.45–0.50; lb 2.45–2.80/2.75–2.90; mew 1.10–1.25/1.35–1.45; mith 0.15–0.20/0.20; mpw 0.60–0.70/0.70–0.75; mth 0.30–0.40/0.35; pl 0.50–0.55/0.60–0.65; ptbl 0.55–0.70/0.65; ptbmw 0.10–0.15/0.15; rl 0.35/0.40; scl 0.45–0.55/0.55.

#### Type material.

Holotype, ♂– -21.58536/165.79319, Col d’Amieu 500m (3,0 km to gate), 16.11.2008, leg. M. Wanat (MNHN).

Paratypes: 1♂–21037.632'S 165045.830'E, Farino env. Les Grandes Fougéres, rainforest, netting, biting, 12.03.2008 467m, leg. R. Dobosz & T. Blaik; 5915/11630 (BMNH); 1♂,1♀–21.61176/165.75406, Farino, Parc des Grandes Fougéres, 400m Camp de la Houe, 13.11.2008, leg. M. Wanat (MNHW); 1♀–21035.1'S 16504’, Col d’Amieu 490m (3,5 km from gate), 6–7.01.2007 (loc 3), leg. M. Wanat (MNHW); 1♂–-21.58536/165.79319, Col d’Amieu 500m, (3,0 km to gate), 16.11.2008, leg. M. Wanat (MNHW); 1♀–Col d’Amieu, Forestry Station, 18–21 Oct 1978, J.S. Dougdale; malaise trap; N.Z. Arthropod Collection NZAC, Private Bag 92170, Auckland, New Zealand (NZAC); 1♀–21011'S 165016'E, Aoupinié 950–1000m, meteo st.–summit, 8.02.2004, leg. M. Wanat (MNHW); 1♀–Tchamba (Wâo Uni), at day, rainforest, 1.04.2008, leg. T. Blaik (DBUO); 1♂–21000.2'S/16504.6'E, Tchamba (Wâo Uni), 16.01.2007 500–530m, forest, old northern road, leg. M. Wanat (MNHW); 1♂–Mt. Rembai, 700m, 18 Oct 1978, J.C. Watt; beating logging area at nigth; N.Z. Arthropod Collection NZAC, Private Bag 92170, Auckland, New Zealand (NZAC).

#### Etymology.

A toponymical adjective, after Tchamba, where some of the paratypes were collected.

#### Distribution.

New Caledonia (main island, north and south provinces).

### Rasilinus bicolor sp. n.

Taxon classificationAnimaliaColeopteraCurculionidae

http://zoobank.org/3100327F-A4EC-49E3-8D7D-7A0BEAAAED40

Figs 1 , 10 , 19 , 28 , 37 , 49–51 , 77 , 86 , 89 , 94 , 100 , 107 , 114 , 121 , 126 , 131

#### Diagnosis.

Distinguished by the bicolored body with pronotum and basal part of elytra reddish and remaining parts of body black. Pronotal disc with subtriangular mark composed of white scales. Humeral calli strongly prominent.

#### Description.


 Body length (lb) 2.55–2.90 mm.

*Body colour and vestiture* (Fig. 1). Head and rostrum dark brown to black covered with white, suboval (adjoining) and elongate (protruding) scales. Scape orange, darkening to apex; funicle and club darker. Pronotum reddish with subtriangular spot composed of white scales. Elytra bicolored, anterior half reddish, posterior half dark brown to black; intervals with single, protruding, elongate, dark (lighter-colored at apical part) scales. Elytra medially with two small spots on third interval composed of white suboval scales (sometimes hardly visible). Legs dark brown; fore and middle femora with single, white, protruding scales, hind femora with transverse stripe of small, suboval, white scales. Front and middle tibiae with elongate, protruding scales, these are brown on basal part and white on apical two thirds. Tarsi orange.

*Head, rostrum and antennae* (Figs 19, 28, 37). Head capsule transverse (hw/hl *ca.* 1.30). Forehead between eyes flat; eye protruding in dorsal view. Vertex distinctly convex, strongly rough. Eyes rounded, convex, slightly protruding beyond the head outline; longer than half length of head (eyl/hl *ca.* 0.70). Rostrum weakly curved, much longer than wide at apex (rl/arw *ca.* 1.60). Funicle shorter than scape; desmomere 1 elongate, as long as 2+3; 2 elongate, almost as long as 3+4; desmomeres 3–7 slightly longer than wide. Club elongate, *ca.* 2 × longer than wide, as long as last five desmomeres of funicle, compact.

*Pronotum* (Figs 10, 19). Campaniform with weakly rounded apical margin, basal margin slightly medially extended onto scutellum, widely rounded. Widest at base, bpw/pl *ca.* 1.20. Pronotal disc flat in lateral view, with fine depression near apex. Surface with dense punctation, rough; space between punctures matt.

*Elytra* (Figs 10, 19). Widest across humeral calli (el/mew *ca.* 1.50); distinctly narrowed behind humerus, slightly broadened medially, strongly narrowed to apex. Dorsal surface glabrous, shining. Striae with subcircular, well isolated punctures, these becoming less numerous towards apex. Intervals flat, glabrous, with single line of short, protruding scales in two colours: brown on basal four fifths and white on apical fifth.

*Legs* (Figs 49–51). Front and middle legs robust; tibiae bisinuate, short and wide (ptbl/ptbmw *ca.* 4.00); hind tibiae with arched inner margin, distinctly narrowed before apex. Femora with rough surface and distinct, transverse corrugation. Front and middle femora stout with small ventral tooth, surface between tooth and apex with semicircular, deep cavity. Base of hind femora distinctly compressed, slightly twisted. Corrugation on flattened, basal part of legs deeper and parallel horizontally. Claws toothed.

*Abdomen* (Fig. 86). Weakly narrowed, almost as long as maximal width at base. Suture between ventrites 1 and 2 depressed laterally; medially not depressed, only as thin line; sutures between ventrites 2–5 wide, strongly depressed. Last ventrite with widely rounded posterior margin; 2 × as wide as long. Entirely covered with deep punctation and piliform, white setae; surface glabrous, shining. Pygidium of male subquadrate, dorsal surface with sparse punctures. Ventral processes elongate with single, short setae.

*Male terminalia* (Figs 100, 107, 114). Tegminal apodeme short and narrowed. Pedon slightly, regularly tapering toward rounded apex; base fully sclerotized; in lateral view distinctly curved with upturned apex. Basal part of sternite VIII apodeme extended into pair of elongate, slightly curved and tapering processes; Sternite IX with two small, subtriangular sclerites situated at apical part of divided appendices. Tegmen with parameres fused over basal third of length, without intervening membrane.

*Female* (Figs 94, 121, 126, 131). Similar to male in measurements and proportion of body, but slightly larger. Subtriangular white mark on pronotum smaller; two white spots at the middle of elytra absent. Fore tibiae more slender than those of male; subapical part of femora less concave; tooth on fore and middle femora minute. Abdominal tergite VIII subcircular with small punctures at lateral margin; apically with elongate setae some of which are slightly broadened basally. Gonocoxite as in Fig. 121, elongate, slightly constricted medially. Spermatheca stout, short, weakly concave. Apodeme of sternite VIII long, base split into pair of short, acute processes; apical lobe irregular, furnished with elongate setae at anterior angle.

#### Measurements.

Holotype, ♂/paratype, ♀: apw 0.50/0.55; arw 0.25/0.30; bew 1.15/1.35; bpw 0.65/0.75; el 1.75/2.00; eyl 0.25/0.30; frw 0.15/0.15; hl 0.35/0.40; hw 0.25/0.30; lb 2.55/2.90; mew 1.15/1.35; mith 0.20/0.25; mpw 0.65/0.75; mth 0.30/0.35; pl 0.55/0.60; ptbl 0.60/0.70; ptbmw 0.15/0.15; rl 0.40/0.45; scl 0.55/0.55.

#### Type material.

Holotype, ♂–20°33'S/164°48'E, 20-50 m., Cascade de Tao, humid forest along stream, 01.02. 2004, leg. M. Wanat (MNHN).

Paratypes: 1♀– 20°23.9'S/164°31.9'E, Mandjélia (summit), 12.01.2007, 750-780 m, beating, montane forest, leg. M. Wanat & R. Dobosz (MNHW); 1♀– 20°33'S/164°46'E, Mt. Panié, 500–1000 m, E track, humid forest, 03.02.2004, leg. M. Wanat (MNHW).

#### Etymology.

This specific epithet is derived from the Latin prefix *bi*– (two), and noun *color* (color, pigment) and refers to the bicolored body. A noun in apposition.

#### Distribution.

New Caledonia (main island, north province).

### Rasilinus bifurcatus sp. n.

Taxon classificationAnimaliaColeopteraCurculionidae

http://zoobank.org/B42F2DD0-A97C-4E1E-B773-CCB252CC9B7B

Figs 2 , 11 , 20 , 29 , 38 , 52 ‒54, 78, 87, 99, 101, 108, 115

#### Diagnosis.

The species can be distinguished from other species by the following combination of features: rostrum short and wide; eyes strongly convex laterally; lateral line of temples longer than length of eyes, distinctly divergent. Last abdominal ventrite apically with two shallow grooves bearing elongate setae. Parameroid lobes of tegmen separate to base. Internal sac of aedeagus with two, elongate, sclerites. Most similar to *Rasilinus longulus* sp. n. but can be easily distinguished by toothed tarsal claw (unarmed in *Rasilinus longulus* sp. n.), stronger convex eyes–hw/hl = 1.00 (*Rasilinus longulus* sp. n.–hw/hl = *ca.* 1.20) and shorter rostrum–rl/arw = 1.50 (*Rasilinus longulus* sp. n.–rl/arw = 1.70).

#### Description.


 Body length (lb) *ca.* 3.20 mm.

*Body colour and vestiture* (Fig. 2). Head, pronotum and elytra dark brown to black; rostrum and legs reddish, apex dark; apical part of tibiae darkening. Antennal scape orange; funicle and club darker. Dorsal surface of rostrum covered with suboval, white scales, middle of rostrum with asetose line. Ventral side of rostrum at base and prosternum covered densely with suboval, white scales. Legs with mixed dark and white elongate scales; hind femora with indistinct transverse stripe composed of small, suboval, white scales.

*Head, rostrum and antennae* (Figs 20, 29, 38). Head capsule subquadrate (hw/hl *ca.* 1.00). Forehead flat. Eyes convex, strongly protruding dorsally, shorter than half length of head (eyl/hl *ca.* 0.45). Rostrum weakly curved; longer than wide at apex (rl/arw *ca.* 1.50). Funicle shorter than scape; desmomere 1 strongly broadened, *ca.* 2 × as long as maximal width, as long as 2+3; 2 elongate, longer than 3; 3 longer than wide; 4–7 subquadrate. Club elongate, more than 2 × longer than wide, almost as long as desmomeres 2–7; compact.

*Pronotum* (Figs 11, 20). Shorter than wide (mpw/pl *ca.* 1.20); apical margin straight; base weakly rounded. Widest at middle; anterior half narrower than base. Pronotal disc, when viewed laterally, more sloping towards head than elytra. Surface strongly rough with dense punctation, space between punctures with microsculpture, matt.

*Elytra* (Figs 11, 20). Widest behind humeral calli, elongate (el/mew *ca.* 1.60), from middle of length strongly narrowed to apex. Dorsal surface slightly shining. Striae with suboval, well isolated punctures, in distal two-thirds of length becoming less numerous towards apex. Intervals weakly convex across whole length.

*Legs* (Figs 52–54, 78). Front and middle legs elongate; tibiae straight, slender (ptbl/ptbmw *ca.* 5.30). Hind tibiae slender, regularly curved. Front femora glabrous, without tooth; middle femora with small tooth. Claws with obtuse teeth.

*Abdomen* (Fig. 87, 99). Weakly narrowed, slightly longer than wide at base. Sutures between ventrites 2–5 wide and strongly depressed. Last ventrite almost 2 × wider than long; surface with sparse punctures and elongate setae; lateral margin along two–thirds of length with distinctive, sharp edges, apically with two shallow grooves, each bearing few elongate setae; apical margin widely rounded. Pygidium of male subquadrate, dorsal surface with single, sparse punctures. Ventral processes broad, stout, subquadrate with concentration of elongate setae in the middle and on apical margin.

*Male terminalia* (Figs 101, 108, 115). Aedegal pedon almost as long as apodemes; regularly narrowed, strongly constricted apically with rounded apex; basal part of pedon completely sclerotised. In lateral view regularly curved with slightly upturned apex. Internal sac with irregular transfer apparatus and two elongate, acute processes. Tegmen with parameroid lobes shorter than apodeme; parameroid lobes completely bilobed, entirely surrounded by membrane; apical part of membrane with distinct, elongate setae. Sternite VIII with elongate, stout apodeme; basal part of apodeme enlarged. Sternite IX with two highly visible, irregular sclerites.

*Female*. Unknown

#### Measurements.

Holotype, ♂: apw 0.60; arw 0.30; bew 1.30; bpw 0.75; el 2.15; eyl 0.25; frw 0.20; hl 0.55; hw 0.55; lb 3.20; mew 1.35; mith 0.25; mpw 0.80; mth 0.50; pl 0.65; ptbl 0.80; ptbmw 0.15; rl 0.45; scl 0.60.

#### Type material.

Holotype, ♂– 22°11.0'S/165°17.6'E, Auopinié, 850–900 m., 18.01.2007, forest, leg. M. Wanat & R. Dobosz (MNHN).

#### Etymology.

From the Latin prefix *bi*– (two), and noun *furca* (fork, two-pronged), refers to the two characteristic, elongate sclerites in the internal sac of aedeagus. An adjective.

#### Distribution.

New Caledonia (main island, north province).

### Rasilinus bimaculatus sp. n.

Taxon classificationAnimaliaColeopteraCurculionidae

http://zoobank.org/1C320791-3DE2-4EA7-89F1-58FACEC1E9B3

Figs 3 , 12 , 21 , 30 , 39 , 46 , 55 ‒57, 79, 88, 102, 109, 116

#### Diagnosis.

It differs from other members of the new genus in having two characteristic spots in the middle of elytra. Body elongate; head behind eyes wide and stout with parallel lateral margin of vertex, eyes hardly concave; claws strongly curved with prominent, acute basal tooth.

#### Description.


 Body length (lb) *ca.* 3.30 mm.

*Body colour and vestiture* (Fig. 3). Pronotum and elytra dark brown; dorsal surface of rostrum covered with suboval, white scales, middle of rostrum with asetose line; underside of rostrum with white, elongate scales. Pair of white spots at the middle of elytra (at third intervals) composed of small, strongly imbricate, suboval, white scales; intervals with line of protruding, elongate, dark and white scales. Femora brown with darker base; tibiae and tarsi dark orange. Antennae uniformly brown with brighter club. Hind femora with distinct transverse stripe composed of small, suboval, white scales.

*Head, rostrum and antennae* (Figs 21, 30, 39). Head capsule subquadrate (hw/hl *ca.* 1.00). Forehead flat. Eyes strongly convex, distinctly protruding above margin of head in dorsal and lateral views; half as long as head (eyl/hl *ca.* 0.50). Vertex with distinctly, well isolated punctures. Rostrum short (rl/arw *ca.* 1.30), weakly curved. Funicle shorter than scape; desmomere 1 elongate, almost as long as 2–4; 2 slightly longer than 3; 3 subquadrate; 4–7 wider than long. Club more than 2 × longer than wide; as long as desmomeres 2–7.

*Pronotum* (Figs 12, 21). Slightly shorter than wide (mpw/pl *ca.* 1.15); sides parallel, apically strongly narrowed; base bisinuate. Pronotal disc, when viewed laterally, more sloping towards head than elytra. Surface strongly rough with dense punctation, space between punctures with microsculpture, matt.

*Elytra* (Figs 12, 21). Widest across weakly protruding humeral calli, distinctly elongate (el/mew *ca.* 1.75), apical two thirds regularly tapering to apex. Dorsal surface glabrous, shining. Striae with suboval, well-isolated punctures, from one third of length becoming less numerous towards apex. Intervals flat across entire length.

*Legs* (Figs 55–57, 79). Front and middle femora stout, weakly narrowed basally; fore femora with very small, middle with distinct tooth on ventral margin. Tooth on hind femora sharp and narrow. Front tibiae bisinuate, broadened apically; middle tibiae elongate, weakly bisinuate, not broadened apically; hind tibiae regular curved, slightly narrowed apically. Claws strongly curved with prominent, acute, basal tooth.

*Abdomen* (Fig. 46, 88). Weakly longer than maximal width, strongly narrowed to apex. First suture obsolete medially; sutures between ventrites 2–5 wide and strongly depressed. Last ventrite almost 2 × as wide as long with distinct, suboval cavity at middle; margin of cavity at anterior half with strongly elongate, bright setae, similar setae (but more sparse) on whole apical margin of last ventrite. Pygidium of male longer than wide, dorsal surface with sparse punctation; apical margin with elongate setae; ventral processes suboval with elongate setae at basal part and apical margin.

*Male terminalia* (Figs 102, 109, 116). Aedegal pedon shorter than apodemes, basally fully sclerotised and broadened, medially slightly narrowed; apex truncate, widely rounded. From lateral view distinctly broadened medially. Internal sac with single kidney shaped sclerite. Apodeme of sternite VIII strongly broadened and curved apically in lateral view, basal part arrow shaped; sternite IX with pair of clearly visible, subtriangular sclerites. Apodeme of tegmen broadened apically, longer than parameroid lobe, which is not completely divided.

*Female*. Unknown

#### Measurements.

Holotype, ♂: apw 0.60; arw 0.35; bew 1.35; bpw 0.85; el 2.30; eyl 0.25; frw 0.20; hl 0.50; hw 0.55; lb 3.35; mew 1.35; mith 0.25; mpw 0.80; mth 0.45; pl 0.70; ptbl 0.75; ptbmw 0.15; rl 0.45; scl 0.60.

#### Type material.

Holotype, ♂– No. 1194, 22°24.0'S × 164°31'E, 580 m., Mandjelia, lower creek, 12–13 Dec 2004, rainforest, leg. G.B. Monteith, beating (MNHN).

#### Etymology.

From the Latin prefix *bi*– (two), and noun *macula* (spot), refers to the two characteristic spots in the middle of the elytra. An adjective.

#### Distribution.

New Caledonia (main island, north province).

### Rasilinus grandidens sp. n.

Taxon classificationAnimaliaColeopteraCurculionidae

http://zoobank.org/60A7AAB7-49DD-4195-B585-E09B08AF96E5

Figs 4 , 13 , 22 , 31 , 40 , 57 ‒60, 80, 93, 95, 117, 122, 127, 132

#### Diagnosis.

This species is unique among members of the genus in having a blunt, large tooth on the basal part of the tarsal claws. Forehead and basal part of rostrum with suboval, white scales. Elytral striae composed of irregular punctures, from third striae outwards punctures fused into irregular line. Apical part of suture with stripe composed of fine, pale scales.

#### Description.


 Body length (lb) *ca.* 2.60 mm.

*Body colour and vestiture* (Fig. 4). Body generally blackish; legs dark brown; antennal scape orange, darkening toward apex; funicle and club dark brown. Elytral intervals with line of protruding, elongate, dark scales. First interval apically with indistinct line composed of white, minute, suboval scales. Hind femora with distinct transverse stripe composed of small, suboval, white scales.

*Head, rostrum and antennae* (Figs 22, 31, 40). Head transverse (hw/hl *ca.* 1.50). Forehead flat. Eyes convex, weakly protruding above margin of head in lateral and dorsal views, longer than half total length of head (eyl/hl *ca.* 0.80). Vertex without punctures, rough, matt. Rostrum less than twice its apical width (rl/arw *ca.* 1.60), weakly curved; dorsal part with two types of scales: one short and adjacent and other elongate, strongly protruding. Funicle shorter than scape; desmomere 1 enlarged, elongate, as long as 2–4; 2 longer than 3; 3–5 subquadrate; 6–7 wider than long. Club 2 × as long as wide, as long as desmomeres 2–7.

*Pronotum* (Figs 13, 22). Subisodiametric (mpw/pl *ca.* 1.10); slightly broadened to base, apically distinctly narrowed; base slightly bisinuate. Surface distinctly rough, matt, with short scales directed forwards.

*Elytra* (Figs 13, 22). Elongate (el/mew *ca.* 1.70), widest at about one–third of length, slightly tapering toward apex. Striae with subcircular, distinctive punctures, those of striae 1–2 fused into irregular line on their apical two thirds; striae 3–10 without distinctive punctures formed by shallow line. Intervals convex.

*Legs* (Figs 57–60, 80). Femora slender, fore unarmed, middle with small tooth, hind with relatively small and narrowed tooth. Tibiae elongate, slender, fore and middle straight, hind tibiae distinctive curved and slender, narrowed subapically. Claws strongly curved with broadened, acute basal tooth.

*Abdomen* (Fig. 93). Elongate, 1.30 × longer than wide. First suture obsolete along entire length, visible as thin line. Sutures between ventrites 2–5 distinctive, visible as strongly depressed line. Last ventrite subtriangular, widely rounded apically. Pygidium of female subtriangular, rounded apically; surface asetose with sparse punctures.

*Female terminalia* (Figs 95, 122, 127, 132). Sternite VIII with bifurcate base and pair of subquadrate lobes; apodeme strongly elongate. Abdominal tergite VIII subquadrate with parallel lateral margin. Apical margin widely rounded with very long, acute setae. Surface with sparse punctures. Ovipositor as in Fig. 122. Spermatheca as in Fig. 127.

*Male*. Unknown.

#### Measurements.

Holotype, ♀: apw 0.50; arw 0.25; bew 1.05; bpw 0.60; el 1.75; eyl 0.25; frw 0.15; hl 0.30; hw 0.45; lb 2.60; mew 1.05; mith 0.20; mpw 0.60; mth 0.25; pl 0.55; ptbl 0.60; ptbmw 0.10; rl 0.40; scl 0.45.

#### Type material.

Holotype, ♀– 22°11'S/165°17'E, Aoupinie, 900–950 m., gate–meteo st., 8.02.2004, leg. M. Wanat (MNHN).

#### Etymology.

The specific epithet derived from the Latin adjective *grandis* (big, large) and the noun *dens* (a tooth) and refers to the distinctly toothed claws. A noun in apposition.

#### Distribution.

New Caledonia (main island, north province).

### Rasilinus longulus sp. n.

Taxon classificationAnimaliaColeopteraCurculionidae

http://zoobank.org/A456689F-20D7-4475-968F-274CE3101AE9

Figs 5 , 14 , 23 , 32 , 41 , 61 ‒63, 81, 96, 123, 128, 133

#### Diagnosis.

The following combination of characters allows this species to be distinguished from its congeners: rostrum elongate, distinctly curved laterally; ventral margin of fore and middle femora glabrous, without teeth; tarsal claws untoothed; base of sternite VIII in female as enlarged plate with distinctive, deep recess.

#### Description.


 Body length (lb) *ca.* 3.50 mm.

*Body colour and vestiture* (Fig. 5). Pronotum and elytra dark brown to blackish; head, rostrum and legs brighter; scape yellowish, funicle and club darker, brown. Dorsal surface of rostrum and outer margin of fore and middle tibiae with elongate, white scales. Hind femora with distinct transverse stripe composed of small, suboval, white scales. Pronotum entirely covered with short, indistinct, brown scales. Elytral intervals with line of protruding, elongate, dark scales.

*Head, rostrum and antennae* (Figs 23, 32, 41). Head subquadrate (hw/hl 0.90–1.00). Forehead flat. Eyes weakly convex laterally; protruding above margin of head in lateral view; half as long as head (eyl/hl; *ca.* 0.50); lateral margin of head strongly narrowed from base to hind margin of eyes. Vertex with sparse punctation, space between punctures rough, matt. Rostrum with length nearly 2 × its apical width (rl/arw *ca.* 1.70), curved laterally; dorsal part with two types of scale: one short and adjacent and other elongate, strongly protruding; middle of rostrum with asetose longitudinal line. Funicle shorter than scape; desmomere 1 *ca.* 1.50 × as long as 2; 2 almost 2 × longer than 3; 3–7 slightly longer than wide. Club 2 × as long as it is wide, as long as desmomeres 3–7.

*Pronotum* (Figs 14, 23). Slightly longer than wide (mpw/pl *ca.* 1.15); side almost parallel, subapically distinctly narrowed; base and apical margin straight. Surface distinctly rough, matt.

*Elytra* (Figs 14, 23). Elongate (el/mew *ca.* 1.65), widest at about one–third of length; distinctly narrowed to apex. Striae with small, elongate punctures; apically with punctures disappearing, forming indistinct line. Intervals rugose, flat.

*Legs* (Figs 61–63, 81). Femora robust; front and middle with ventral margin glabrous, without tooth. Tibiae elongate, slender; fore and middle straight, hind regularly curved. Claws untoothed, only broadened basally.

*Abdomen*. Elongate, *ca.* 1.20 × longer than wide. First suture obsolete along whole length. Sutures between ventrites 2–5 strongly depressed medially. Last ventrite subtriangular, widely rounded apically.

*Female terminalia* (Figs 96, 123, 128, 133). Abdominal tergite VIII massive, distinctly longer than wide; apex widely rounded, covered with long setae. Ovipositor slender, almost straight. Spermatheca L–shaped. Sternite VIII with long, thin apodeme; base with enlarged, suboval plate, deeply recessed medially.

*Male*. Unknown.

**Measurements.** Holotype, ♀/paratypes, ♀: apw 0.65/0.60–0.65; arw 0.35/0.30–0.35; bew 1.50/1.45–1.50; bpw 0.85/0.80–0.85; el 2.40/2.30–2.35; eyl 0.25/0.25; frw 0.15/0.15; hl 0.50/0.50–0.55; hw 0.50/0.45–0.50; lb 3.50/3.55–3.60; mew 1.50/1.45–1.50; mith 0.25/0.25; mpw 0.85/0.80–0.85; mth 0.45/0.45; pl 0.75/0.75; ptbl 0.80/0.80; ptbmw 0.10/0.10; rl 0.60/0.60; scl 0.65/0.60–0.65.

#### Type material.

Holotype, ♀– 21°11'S/165°17'E, Aoupinie, 850–900 m., 18.01.2007, forest, leg. M. Wanat & R. Dobosz (MNHN).

Paratype: 1♀, No. 8683, 20°58'S × 165°17'E, 500 m, Pic d’Amoa, N slopes, 10–24 Nov. 2001, leg C. Burwell & G. Monteith, malaise trap (QM); 1♀, No. 12003, 21°35'S × 165°51'E, 780 m, Mt. Rembai, top junction, 19 Dec 2004, leg. G. Monteith, beating, rainforest (QM).

#### Etymology.

The specific epithet is derived from the Latin adjective *longus* (long) and diminutive ending –*ulus* and refers to the elongate rostrum.

#### Distribution.

New Caledonia (main island, north and south provinces).

### Rasilinus subgemellus sp. n.

Taxon classificationAnimaliaColeopteraCurculionidae

http://zoobank.org/F4F6F30A-9F12-4744-ADA4-FB99D222770B

Figs 6 , 15 , 24 , 33 , 42 , 47 , 64 ‒66, 82, 89, 103, 110, 117

#### Diagnosis.

The species is unique within the genus in having a pair of small nodules at the middle of the first ventrite of male; rostrum with distinct median keel; regularly rounded lateral margin of pronotum and parameroid lobes of tegmen with a pair of very short, sclerotised processes.

#### Description.


 Body length (lb) *ca.* 3.60 mm.

*Body colour and vestiture* (Fig. 6). Pronotum, elytra and tibiae dark brown to blackish; femora brown with darker base and apex. Scape of antennae orange, funicle and club darker. Dorsal part of rostrum covered with white, suboval scales. Tarsus with strongly elongated, white scales, sometimes much longer than length of tarsomeres. Hind femora with distinct, transverse, wide stripe composed of small, suboval, white scales. Intervals with line of elongate, strongly protruding, dark scales. Abdomen dark orange, apical margins of ventrites and pair of nodules much darker.

*Head, rostrum and antennae* (Figs 24, 33, 42). Head subquadrate (hw/hl *ca.* 1.10). Forehead slightly concave. Eyes relatively small, weakly convex laterally; slightly protruding above margin of head in lateral view; approximately as long as half-length of head (eyl/hl *ca.* 0.40). Vertex distinctly convex, with irregular, dense punctation, strongly scabrous; lateral margin broadened, widely rounded near base. Rostrum almost as long as it is double width at apex (rl/arw *ca.* 1.85), as long as head, weakly curved laterally; medially with distinct, protruding keel. Funicle shorter than scape; desmomere 1 strongly elongate, as long as 2–4 taken together; 2 slightly longer than 3; 3–7 slightly longer than wide. Club 2 × than wide, as long as desmomeres 3–7.

*Pronotum* (Figs 15, 24). Distinctly wider than long (mpw/pl *ca.* 1.25); laterally regularly rounded, narrowed subapically. Surface strongly rough, matt.

*Elytra* (Figs 15, 24). Elongate (el/mew *ca.* 1.55), widest across humeral calli, weakly narrowed to middle of length, strongest apically. Striae narrow, composed of small punctures, slightly concave only at apical part. Intervals wide, flat, strongly rugose, shining.

*Legs* (Figs 64–66, 82). Fore and middle legs elongate, slender; femora with tooth at ventral side; tibiae slightly bisinuate, narrowed subapically. Hind tibiae regularly curved. Claws untoothed, basally distinctly, regularly broadened.

*Abdomen* (Fig. 47, 89). Slightly longer than wide. First ventrite with two distinct nodules; suture distinct laterally, medially obsolete. Sutures between ventrites 2–5 strongly depressed. Last ventrite wider than long; apical margin straight. Pygidium of male subquadrate, dorsal surface with sparse punctation. Apical margin with elongate setae. Ventral processes relatively small; with few, sparse setae.

*Male terminalia* (Figs 103, 110, 117). Aedegal pedon longer than apodemes, basal part unsclerotised, extended into pair of processes; apex strongly narrowed, acuminate. From lateral view curved irregularly with slightly upturned apex. Tegmen with subcircular tegminal ring; tegminal apodeme thin and short; parameroid lobes indistinct, composed of two fully separated, short processes, each surrounded by thin membrane. Basal part of sternite VIII irregularly bifurcate; hemisternites on sternite IX irregular, well visible.

*Female*. Unknown.

#### Measurements.

Holotype, ♂: apw 0.70; arw 0.35; bew 1.60; bpw 0.95; el 2.50; eyl 0.30; frw 0.20; hl 0.65; hw 0.60; lb 3.60; mew 1.60; mith 0.25; mpw 1.00; mth 0.35; pl 0.80; ptbl 0.85; ptbmw 0.15; rl 0.65; scl 0.70.

#### Type material.

Holotype, ♂– Loyality Is., Lifu, nr. We(Oue), 2–35m, 26–28.III.1968; T.C. Maa, Collector, Bishop; N.Z. Arthropod Collection, private bag 92170, Auckland, New Zealand (MNHN).

#### Etymology.

The specific epithet is derived from the Latin prefix *sub*– (on the lower side, beneath) and Latin adjective *gemellus* (paired, double) and refers to the pair of nodules situated on the first segment of the male abdomen.

#### Distribution.

New Caledonia (Lifou Island).

### Rasilinus subnodulus sp. n.

Taxon classificationAnimaliaColeopteraCurculionidae

http://zoobank.org/B1AE3E3A-E2E7-402C-AE85-C7E66FD52DF9

Figs 7 , 16 , 25 , 34 , 43 , 48 , 67 ‒69, 83, 90, 97, 104, 111, 118, 124, 129, 134

#### Diagnosis.

The species is unique within the genus in having a strongly convex pronotum and elytral disc; large tubercle in the middle of first ventrite in male; internal sac with characteristic sclerite with two elongate lobes; tegmen with parameroid lobes completely divided into two, thin parameres. Spermatheca of female slender and strongly curved; apex of sternite VIII T–shaped with well-developed, split almost to base, hemisternites.

#### Description.


 Body length (lb) 3.20–3.60 mm.

*Body colour and vestiture* (Fig. 7). Head, pronotum and elytra dark brown to blackish ventrally and dorsally. Legs varying from uniformly reddish to blackish with lighter medial part of femora or blackish basal part. Scape of antennae orange, funicle and club darker. Tarsi same colour as tibiae, sometimes last tarsomere lighter.

*Head, rostrum and antennae* (Figs 25, 34, 43). Head subquadrate (hw/hl ca 1.10). Forehead flat, wide (frw/hw *ca.* 0.45). Eyes distinctly convex laterally; length approximately half length of head (eyl/hl *ca.* 0.45). Vertex with distinctive, deep punctation, surface rough. Rostrum as long as head or longer (rl/hl 1.00–1.20), distinctly curved, punctures only laterally, glabrous medially and matt on apical third. Funicle as long as scape; desmomere 1 as long as 1–2 taken together; 2 longer than 3; 3–7 subquadrate. Club 2 × than wide, as long as desmomeres 3–7.

*Pronotum* (Figs 16, 25). Subquadrate (mpw/pl = 1.00–1.07); dorsally equilateral at base, strongly narrowed at apical half. Disc distinctly convex. Surface distinctly rough, densely punctate, matt.

*Elytra* (Figs 16, 25). Elongate (el/mew = 1.40–1.58), widest across humeral calli, regularly narrowed from base to apex. In lateral view distinctly convex. Striae narrow, composed of small punctures, becoming evidently smaller to obsolescent from middle of disc towards apex. Intervals wide, flat, glabrous, slightly shining.

*Legs* (Figs 67–69, 83). Fore tibiae distinctly narrowed apically; femora with minute tooth near midlength. Mid legs similar, tibiae weakly narrowed apically; femora with distinct tooth beyond midlength. Hind tibiae regularly curved. Claws weakly curved, basally with broadly rounded tooth and two pairs of elongate setae: one on bottom and second on upper side of the claw.

*Abdomen* (Figs 48, 90). Elongate, *ca.* 1.20 × as long as maximum width at base. First ventrite with one distinct median nodule. Sutures between ventrites 2–5 strongly depressed. Last ventrite wider than long, slightly narrowed apically. Pygidium of male longer than wide, apical margin straight with elongate setae; inner folds distinct, asetose.

*Male terminalia* (Figs 104, 111, 118). Aedegal pedon longer than apodemes; lateral margins parallel with weak concavity at middle of length; apex widely rounded. In lateral view nearly straight basally, curved more strongly distally, slightly narrowed apically; basal part sclerotised, narrowed, extended into prominent, acute process. Endophallus with well visible transfer apparatus with pair of prominent process. Tegmen well sclerotised, incised to the base parameroid lobes; tegminal apodeme stout, broadened apically, as long as diameter of tegminal ring. Basal part of sternite VIII arrow–shaped, hemisternites on sternite IX elongate, well visible.

*Female* (Figs 97, 124, 129, 134). Similar to male body shape but slightly larger. Rostrum more elongate than in male (rl/arw 1.50–1.70, in male *ca.* 1.40). First ventrite of abdomen without nodule, glabrous; last ventrite widely rounded. Abdominal tergite VIII wider than long with incrassate outer margin and elongate setae; surface sparsely punctate and with isolated short setae; apical margin rounded. Spermatheca strongly curved. Gonocoxite stout, conical. Apodeme of sternite VIII split into pair of short, acute processes connected with well-developed hemisternites.

#### Measurements.

Holotype, ♂: apw 0.65; arw 0.35; bew 1.45; bpw 0.80;el 2.20; eyl 0.25; frw 0.25; hl 0.50; hw 0.55; lb 3.50; mew 1.45; mith 0.25; mpw 0.80; mth 0.40; pl 0.75; ptbl 0.90; ptbmw 0.15; rl 0.50; scl 0.60.

Paratypes, ♂/♀: apw 0.65–0.70/0.75; arw 0.35/0.35–0.40; bew 1.45–1.60/1.60–1.65; bpw 0.80–0.90/0.90–1.00; el 2.20–2.40/2.25–2.60; eyl 0.25/0.25–0.30; frw 0.25/0.20–0.25; hl 0.40–0.55/0.50–0.60; hw 0.55–0.60/0.55–0.60; lb 3.25–3.50/3.50–3.60; mew 1.45–1.60/1.60–1.65; mith 0.25/0.25; mpw 0.80–0.90/0.90–1.00; mth 0.40–0.45/0.40–0.45; pl 0.75–0.90/0.75–0.80; ptbl 0.80–1.00/0.95–1.00; ptbmw 0.150.20/0.15–0.20; rl 0.45–0.50/0.60–0.65; scl 0.55–0.65/0.65–0.70.

#### Type material.

Holotype, ♂– 21°11.0'S 165°17.6'E; Aoupinié, 700–900 m.; 18.01.2007, forest; leg. R. Dobosz & M. Wanat (MNHN).

Paratypes: 1♀–No. 11975; 20°58'S×165°17'E, 480m.; Pic d’Amoa, north slope; 3 Jan 2005, rainforest; G. Monteith, beating (QM); 1♀–No. 11986; 21°09'S×165°19'E, 500m.; Aoupinié, lower east road; 17Dec2004; G. Monteith, beating, rainforest (QM); 1♀–No. 11984; 21°09'S×165°19'E, 500m.; Aoupinié, sawmill; 2 Jan 2005; G. Monteith, beating, rainforest (QM); 1♀–No. 11981; 21°09'S×165°19'E, 500m.; Aoupinié, sawmill; 17 Dec 2004; G. Monteith, MV light, rainforest (QM); 1♀–450–550 m., 5.2.63, Kuschel leg.; Col. d.Rousseltes (NZAC); 2♀♀–21°00.3'S 165°14.9'E; Tchamba (Wâo Uni); 15.01.2007, refuge, 400 m.; night coll. (lamp & beating) leg. M. Wanat & R. Dobosz (MNHW); 1♀–21°10.8'S 165°18.1'E; Aoupinié, 650 m.; 18.01.2007, night beating; leg. M. Wanat (MNHW); 3♂♂–21°11.0'S 165°17.6'E; Aoupinié, 700–900 m.; 18.01.2007, forest; leg. R. Dobosz & M. Wanat (MNHW); 1♀–21.14890/165.32348; Aoupinié (refuge), 400 m.; beating rainforest; 29.11.2008; leg. M. Wanat (MNHW); 1♀–20.95280/165.29135; Pic d’Amoa (Povila), 400 m., rainforest; 22.11.2008; leg. M. Wanat (MNHW); 1♂–21°00.3'S 165°14.9'E; Tchamba (Wâo Uni); 1.04.2008, day, rainforest; leg. T. Blaik (DBUO).

#### Etymology.

The specific epithet is derived from the Latin prefix *sub*– (on the lower side, beneath) and Latin noun *nodulus* (knob, tubercle) and refers to the distinct nodule situated on the first segment of the male abdomen. A noun in apposition.

#### Distribution.

New Caledonia (main island, north province).

### Rasilinus virgatus sp. n.

Taxon classificationAnimaliaColeopteraCurculionidae

http://zoobank.org/37FD7535-D457-46C2-8508-4DB6070BFF02

Figs 9 , 18 , 27 , 36 , 45 , 73 ‒75, 85, 92, 106, 113, 120

#### Diagnosis.

The following combination of characters allows this species to be distinguished from its congeners: head capsule almost quadrate; rostrum small, shorter than head capsule; elytra with stripe composed of pale scales at apical part of suture; tegmen with membranous parameroid lobes and dorsal part of tegminal ring bearing a pair of short, broadly rounded sclerotised lobes; aedeagus with complex transfer apparatus in endophallus; male pygidium with enlarged ventral lobes, occupying almost half area of inner part of pygidium.

#### Description.


 Body length (lb) *ca.* 3.00 mm.

*Body colour and vestiture* (Fig. 9). Pronotum and elytra dark brown to blackish, head and rostrum lighter in colour. Legs uniformly brown with brighter tarsal claws. Rostrum covered with pale, suboval scales. Antennae with light brown scape and darkening funicle and club. Elytra with single line of erect, elongate, dark scale on each interval, additional surface entirely covered with minute, hardly visible, short, piliform, recumbent scales.

*Head, rostrum and antennae* (Figs 27, 36, 45). Head almost subquadrate (hw/hl *ca.* 1.10). Forehead flat. Eyes distinctly protruding above margin of head in lateral view; more than half as long as head (eyl/hl 0.67). Lateral margin of head slightly broadened to base. Vertex rough dorsally, with distinct punctation laterally. Rostrum short and robust, slightly shorter than head capsule (rl/hl *ca.* 0.90), *ca.* 1.60 × longer than maximum width (rl/arw), distinctly curved. Scape almost as long as funicle and club combined; first desmomere elongate, as long as 2–4; desmomere 2 broadened apically, longer than 3; desmomeres 3–7 subcircular, wider than long. Club *ca.* 2.0 × as long as its wide, longer than last six desmomeres.

*Pronotum* (Figs 18, 27). Wider than long (mpw/pl *ca.* 1.35). Strongly narrowed subapically, lateral margin almost entirely distinctly narrowed, broadened only on apical part. Basal margin weakly rounded, apical margin almost straight. Surface with irregular, deep punctures, rough, matt.

*Elytra* (Figs 18, 27). Elongate (el/mew *ca.* 1.55), widest in basal half; humerus not projecting; lateral margins in basal half subparallel, curving and converging apically, apices separate. Striae with suboval, distinct punctures in basal half, apical half with punctures much smaller. Intervals flat, shining, with minute nodules, each bearing elongate, erected seta.

*Legs* (Figs 73–75, 85). Front and middle legs slender; front femora with minute teeth beyond middle, middle femora with tooth more elongate, and hind femora with enlarged tooth with sharp edge; hind femora regularly tapering toward base, fusiform. Front and middle tibiae almost straight, slightly narrowed subapically; hind tibiae regularly curved, narrowed near base. Claws regularly curved, distinctly broadened near base; tooth not developed.

*Abdomen* (Fig. 92). Short, almost as long as its maximum width at base. First suture clearly visible along entire length; sutures 2–4 depressed. Middle area at basal part of first ventrite distinctly depressed. Surface glabrous, shining. Last ventrite subtriangular, almost 2 × wider than long; apical margin regularly rounded. Pygidium of male elongate, *ca.* 1.30 × longer than wide; apical margin straight with numerous elongate setae; ventral lobes enlarged, subquadrate with few, short setae only at apical angles.

*Male terminalia* (Figs 106, 113, 120). Aedeagal pedon as long as apodemes; basal part unsclerotised, extended; lateral margins slightly narrowed; apex with short, prominent process, in lateral view regularly curved. Endophallus with complex apparatus and few free sclerites. Tegminal apodeme broadened apically; dorsal part of tegminal ring with pair of short, broadly rounded processes between the parameroid lobes; parameroid lobes shorter than tegminal apodeme, weakly sclerotised with few, sparse setae. Basal part of sternite VIII with pair of broad, short processes; apodeme elongate, laterally flattened with broadened apex and acute, short teeth beyond middle of length connected with short membrane; hemisternites clearly visible, subcircular.

*Female*. Unknown.

#### Measurements.

Holotype, male: apw 0.55; arw 0.25; bew 1.30; bpw 0.75; el 2.00; eyl 0.30; frw 0.15; hl 0.45; hw 0.50; lb 3.00; mew 1.30; mith 0.20; mpw 0.75; mth 0.50; pl 0.55; ptbl 0.70; ptbmw 0.15; rl 0.40; scl 0.50.

#### Type material.

Holotype, ♂– -22.03188/166.46738, Dzumac Mts 900m, Mt Ouin road junction, night beating, 29.10.2008, leg. M. Wanat (MNHN).

#### Etymology.

The specific epithet is the Latin adjective *virgatus* (striped) and refers to the stripe of pale scales at the apical part of the suture.

#### Distribution.

New Caledonia (main island, south province).

### Key to species

The known species of the new genus *Rasilinus* can be separated as follows:

**Table:** 

1	Body bicoloured, apical part of elytra, legs and head dark brown to black; base of elytra and pronotum reddish (Fig. 1)	***Rasilinus bicolor***
–	Elytra and pronotum uniformly brown to almost black (Figs 2–9)	**2**
2	Elytra with pair of white spots medially (Figs 3, 12, 21)	***Rasilinus bimaculatus***
–	Elytra without any spots	**3**
3	Apical part of elytral suture with a stripe composed of fine, white scales differing from those on elytral disc (Figs 13, 18)	**4**
–	Apical part of elytral suture without distinctive scales forming a stripe	**5**
4	Elytra *ca.* 1.70 × as long as wide; tarsal claws strongly curved with broadened, stout, basal tooth (Fig. 80)	***Rasilinus grandidens***
–	Elytra *ca.* 1.50 × as long as wide; tarsal claws weakly curved, distinctly broadened near base, without tooth (Fig. 85)	***Rasilinus virgatus***
5	Ventral margin of front femora with tooth	**6**
–	Ventral margin of front femora unarmed	**8**
6	Rostrum elongate (rl/arw *ca.* 1.85); median keel on rostrum present (Fig. 33); first abdominal ventrite of male with pair of small, median tubercles (Fig. 47)	***Rasilinus subgemellus***
–	Rostrum shorter (rl/arw = 1.20–1.60); median keel on rostrum absent (Figs 34, 35); first abdominal ventrite of male without median tubercles or with a single large one	**7**
7	Larger species (lb = 3,25–3.60); pronotum subquadrate (mpw/pl = 1.00 –1.07); first abdominal ventrite of male with a large median tubercle (Fig. 48)	***Rasilinus subnodulus***
–	Smaller species (lb = 2.45–2.90); pronotum transverse (mpw/pl = 1.15–1.27); first abdominal ventrite of male without median tubercle	***Rasilinus tchambicus***
8	Middle femora with small tooth (Fig. 53); eyes strongly convex; rostrum stout, shorter than 2 × its maximum wide (Fig. 29); claws with obtuse tooth (Fig. 78)	***Rasilinus bifurcatus***
–	Middle femora unarmed (Fig. 62); eyes weakly convex; rostrum elongate, longer than 2 × its maximum width (Fig. 32); claws only enlarged at base (Fig. 81)	***Rasilinus longulus***

## Discussion

The new genus *Rasilinus* is superficially similar to the genus *Koghicola* Mazur, 2014 but can be easily distinguished by the absence of the prominent processes at the apex of the elytra and normal (not enlarged) trochanters (see Fig. 4 in Mazur 2014b). The new genus, together with genus *Pactola* Pascoe, 1876 and *Koghicola*, has a very characteristic form of the hind legs with strongly broadened femora and curved tibiae. However, the new genus can be easily distinguished from both other genera by the distinctive shape of the femora (clearly visible in dorsal view) (Fig. 76). In *Pactola* the hind femora have a very thin and strongly curved basal half (see Figs 17a–20c in Mazur 2014a), whereas *Koghicola* and *Rasilinus* gen. n. display a weakly curved and more massive base of the hind femora (see Figs 10–12 in Mazur 2014b). Additionally, in *Rasilinus* gen. n. the basal part of the hind femora at its inner side is clearly compressed.

Most known species from the tribe Eugnomini that have been described so far come from New Zealand. From New Caledonia only two genera have been known for many years–the monotypic genus *Callistomorphus* Perroud, 1865 and three species from the genus *Pactola* Pascoe, 1876 (describe at the time as *Macropoda* Montrouzier, 1861, see Mazur 2014a).

Fauna of New Caledonian Eugnomini have not been investigated in greater detail so far. Until now three more species of *Pactola* and one monotypic genus *Koghicola* Mazur, 2014b have been described (Mazur 2014a, b; Mazur and Jezuita 2015), while many others are in preparation (Mazur in prep. and Mazur unpublished data).

A wide distribution of the tribe (from South America to Australian region) with many endemic genera and extremely intra- and interspecies variability causes many problems with redefinition of the tribe. Furthermore, many genera have not been described so far, so phylogeny, zoogeography and origin of the tribe still require further research.

## Supplementary Material

XML Treatment for Rasilinus

XML Treatment for Rasilinus tchambicus

XML Treatment for Rasilinus bicolor

XML Treatment for Rasilinus bifurcatus

XML Treatment for Rasilinus bimaculatus

XML Treatment for Rasilinus grandidens

XML Treatment for Rasilinus longulus

XML Treatment for Rasilinus subgemellus

XML Treatment for Rasilinus subnodulus

XML Treatment for Rasilinus virgatus
